# Large cities lose their growth advantage as countries urbanize

**DOI:** 10.1073/pnas.2529430123

**Published:** 2026-06-25

**Authors:** Andrea Musso, Diego Rybski, Dirk Helbing, Frank Neffke

**Affiliations:** ^a^ Computational Social Science, ETH Zurich, Zurich 8092, Switzerland; ^b^ Complexity Science Hub Vienna, Vienna 1030, Austria; ^c^ Urban Complexity Group, Leibniz Institute of Ecological Urban and Regional Development, Dresden, 01217, Germany; ^d^ Transforming Economies Lab, Interdisciplinary Transformation University Austria, Linz 4040, Austria

**Keywords:** urban growth, cities, urbanization, urban scaling

## Abstract

The share of the world population living in cities with more than one million people rose from 11% in 1975 to 24% in 2025 (our estimates). Will this trend toward greater concentration in large cities continue or level off? We introduce two new city population datasets that use consistent city definitions across countries and over time. The first covers the world between 1975 and 2025, using satellite imagery. The second covers the United States between 1850 and 2020, using census microdata. We find that urban growth follows a characteristic life cycle. In the early stages of a country’s urbanization process, large cities grow faster than smaller ones. At later stages, growth rates equalize across sizes. We use this life cycle to project future population concentration in large cities. Our projections suggest that 38% of the world population will be living in cities with more than one million people by 2100. This estimate is higher than the 33% implied by the well-known theory of proportional growth, but lower than the 42% obtained by extrapolating current trends.

## Significance

Will most people end up living in large cities? This depends on whether large cities tend to grow faster than small ones. Inconsistent city definitions have limited our understanding of this question. We construct two city population datasets with the same definition of cities across countries and over time: a global dataset from satellite imagery (1975–2025) and a U.S. dataset from census microdata (1850–2020). We find that city growth follows a common pattern. In the early stages of a country’s urbanization process, large cities grow fastest. At later stages, cities of all sizes grow at similar rates. Our projections based on this pattern suggest that the rise of large cities will slow down but not stop.

Over the past 50 y, the world population has become increasingly concentrated in large cities. Whether this concentration will continue depends on the relationship between a city’s size and its growth rate—a topic on which the literature is divided.

Theories of increasing returns, such as the New Economic Geography (1, 2) and the evolutionary approach of Pumain et al. (3–5), argue that large cities tend to grow faster than small ones. Large cities are typically more innovative and productive (6, 7), have more educated workforces (8, 9), sit more centrally in trade and knowledge networks (5), and host more advanced economic activities (10, 11). These factors can plausibly confer them a growth advantage. Based on our projection method, even a mild growth advantage—where a 10-fold increase in size corresponds to a 0.7% increase in yearly growth—would result in 47% of the world population living in 1M+ cities by the end of the century.

Theories of proportional growth argue instead that cities of all sizes tend to grow at similar rates (Gibrat’s Law) (12–16). After all, scale also brings costs such as increased congestion (17, 18), crime (19, 20), disease risk (21, 22), and housing expenses (23, 24), and these costs may offset advantages. If city size exhibits no growth advantage, our projections suggest that 33% of the world population will live in 1M+ cities in 2100.

This 14 percentage point gap between the two predictions represents a difference of 1.4 billion people and thus has significant implications for future planning and development. In this paper, we analyze which of these two scenarios is more likely, using two newly built city population datasets. The first dataset, built from satellite-derived grids (25), spans 1975–2025 and covers 99 countries, together representing 94% of the world population. The second, built from census microdata (26–28), spans 1850–2020 and covers the United States (USA) urban system over nearly its entire history.

These datasets substantially extend the spatial and temporal coverage of previous efforts to build such harmonized data (29). Before the advent of satellite imagery and geospatial processing tools, assembling harmonized datasets of city sizes across countries and years was extremely difficult, primarily because national data collection efforts are not designed to produce outputs that are comparable across countries or consistent over time. As a result, most empirical studies of urban growth focused on just a handful of countries over limited time periods. The conclusions of these studies are varied, some supporting proportional growth (13–16) and others not (30–35). Integrating these results has proved difficult due to substantial differences in methodology (36), leaving us with an incomplete understanding of how urban growth evolves over time (37).

Using these new datasets, we sharpen our understanding of temporal trends in urban growth. First, we show that proportional growth and increasing returns are better understood as two phases of the same underlying process, not competing realities. In the early phase of urbanization, large cities enjoy a strong growth advantage, consistent with increasing returns. As an urban system matures, this advantage weakens, and the growth rates of large and small cities converge, consistent with proportional growth. Second, we show that this size–growth relationship translates into systematic changes in the shape of a country’s city-size distribution over time (38–40). When large cities have a growth advantage, the distribution stretches and its rank-size slope [a spline-based analogue of the Zipf exponent (41)] increases. Third, combining this mechanism and a projection method, we project that by 2100, 38% of the world’s population will live in 1M+ cities. This projection lies between the proportional-growth (33%) and increasing-returns (47%) benchmarks, and below an extrapolation of current trends (42%).

## 1. Results

Our city population datasets (Table 1) define cities geographically, as clusters of contiguous built-up areas or regions of high population density (33, 42, 43) (Section 3.2). This definition has three main advantages: i) it dynamically adjusts as urban areas expand; ii) it is consistent across space and time, facilitating robust comparative analyses; and iii) it is unaffected by political boundary changes such as municipal mergers. The trade-off is that this definition captures morphological clusters rather than functional urban areas, so satellite settlements tied to large cities by commuting flows may appear as separate clusters (see *SI Appendix*, section 5 for further discussion).

**Table 1.** Description of the two large-scale city population datasets created for the analysis

Dataset	City-year obs.	Countries	Frequency (years)	Time period
Global cities	1,604,593	99	5	1975–2025
USA cities	26,902	1	10	1850–2020

The Global cities dataset covers 99 countries around the world (amounting to 94% of the world population in 2025) between 1975 and 2025. Its primary source is the GHSL 2023 data package (25). The US cities dataset covers the continental USA between 1850 and 2020. Its primary sources are IPUMS USA (26), IPUMS NHGIS (27), and the Census Place Project (28).

**Fig. 1.**
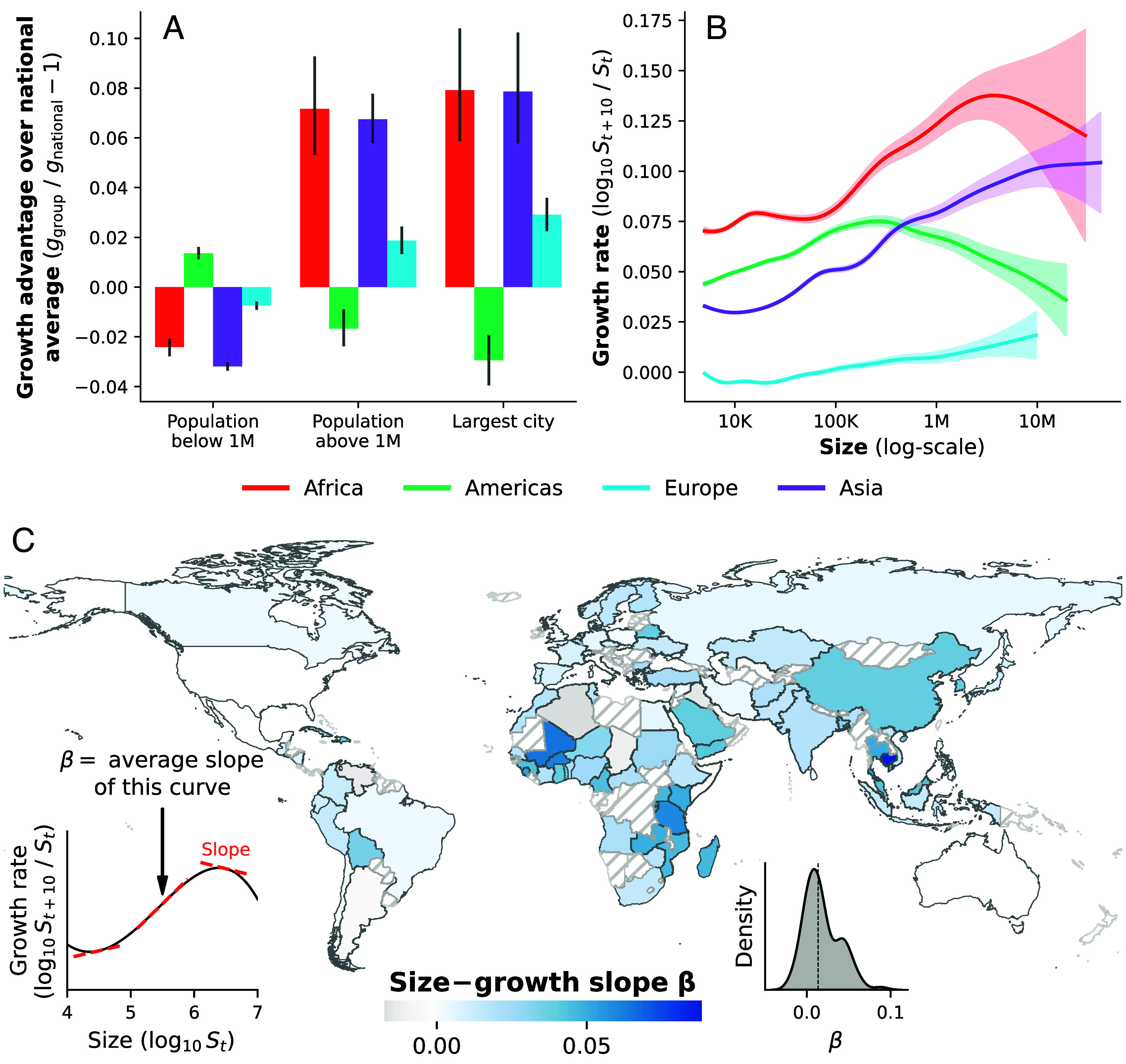
The growth advantage of large cities varies systematically across regions: It is strong in Asia and Africa and weak in Europe and the Americas. (*A*) We compare the average growth rate of a country’s cities, denoted by $g_{\text{national}}$, with the average growth rate of specific subgroups of its cities (e.g., the largest city, 1M+ cities, etc.), denoted by $g_{\text{group}}$. Each bar in the chart shows the ratio $g_{\text{group}}\;/\;g_{\text{national}}-1$ for a given region. (*B*) *Size*-*growth curve* by region. These curves are obtained by fitting a penalized cubic B-spline (λ=100) to the relationship between city log-size in year t and city log-growth between year t and t+10 (Section 3.3.2). (*C*) *Size*-*growth slopes*β by country. β is obtained by first estimating the national size–growth curve [as in panel (*B*)], and then averaging the local slope of this curve across the size spectrum (see *Left Inset* and Section 3.3.2). The map shows the mean value of β between 1975 and 2025; hatched indicates no data. The *Right Inset* shows a kernel density estimate of the distribution of β across countries (dotted line = median).

Our analysis of these datasets reveals substantial global variation in urban growth patterns. Between 1975 and 2025, an average 1M+ city in Asia/Africa outgrew the national average by ∼7% (Fig. 1*A*). In contrast, Europe’s large cities grew modestly faster than the rest (Fig. 1 *A* and *B*), and in the Americas, city growth displayed an inverted-U-shaped trend, with 1M+ cities growing 1.6% slower than their national average (Fig. 1 *A* and *B*).

These patterns are also visible at the country level. Fig. 1*C* maps national size–growth slopes β (definition in the caption of Fig. 1*C*). A positive β indicates that growth increases with size or, in other words, that large cities have a growth advantage. This growth advantage varies significantly across countries, with β ranging from −0.02 to 0.1 (median ≈ 0.012; Fig. 1*C*, *Inset*). Notably, βs are generally higher in Asia and Africa and lower in Europe and the Americas.

**Fig. 2.**
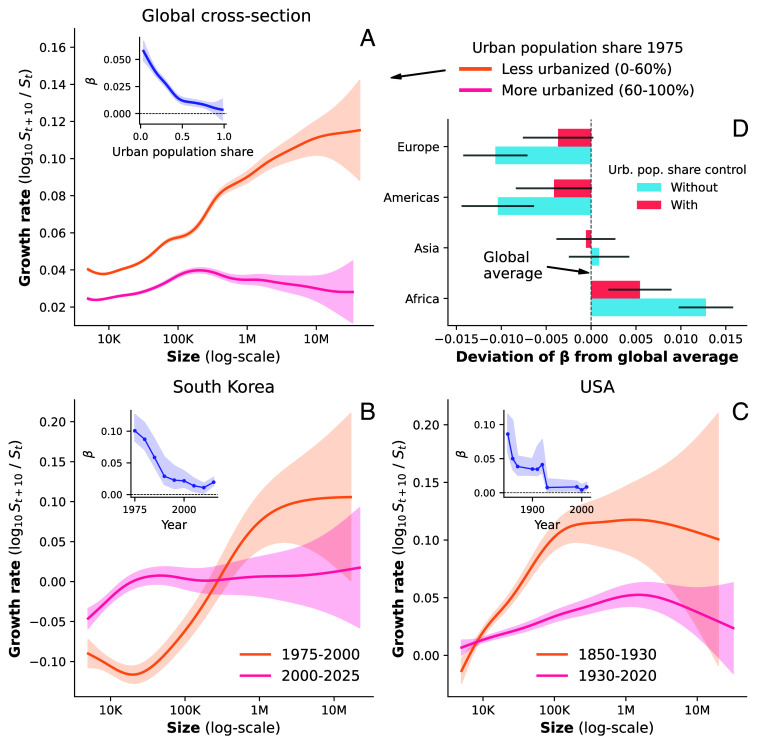
The growth advantage of large cities weakens as countries urbanize. (*A*) Size–growth curve by urbanization group (Section 3.3.2). (*Inset*) β vs. urban population share (fit with a penalized cubic B-spline with λ=100). (*B* and *C*) Size–growth curves in South Korea and the United States across different time periods. (*Insets*) β vs. time (bootstrapped CIs n=1,000). (*D*) Country-level regression with regional dummies ($\beta_{t,\text{country}}∼\delta_{\text{region}}+\text{urban population}{\text{share}}_{t,\text{country}}$). Regional differences in β become significantly smaller once we control for the level of urbanization.

This regional divide can be understood as part of a universal dynamic in which the growth advantage of large cities weakens as a country urbanizes. This inverse relationship is evident in both cross-sectional and longitudinal data (Fig. 2).

Across countries, a lower national share of urban population correlates with higher β (Fig. 2*A*, *Inset* and Table 2). Put differently, growth rates increase rapidly with city size in less urbanized countries, whereas the size–growth relationship is nearly flat in more urbanized ones (Fig. 2*A*). The numbers speak clearly: Between 1975 and 2025, 1M+ cities in more urbanized countries grew at the national average rate, while 1M+ cities in less urbanized ones grew 7.3% faster.

**Table 2.** Association between country urbanization and β

Independent\dependent	Size–growth slope *β*	
Urban population share	−0.049***	−0.049***
	(0.004)	(0.012)
Country fixed effect	No	Yes
Observations	891	891
*R* ^2^	0.168	0.545

^∗^*P* < 0.1; ^∗∗^*P* < 0.05; and ^∗∗∗^*P* < 0.01. Column (1): pooled specification. Column (2): country fixed effects. The sample is the global cities dataset (Table 1).

Within countries, β declines as urbanization rises. A regression with country fixed effects shows that a 20% increase in a country’s urban population share is associated with a ∼0.01 reduction in β (Table 2). This pattern is clearly observed in South Korea and the United States, two countries for which our data cover a large window of the urbanization process. Both countries saw large cities grow substantially faster than smaller ones in the early phase of urbanization, but as urbanization progressed, this advantage largely vanished (Fig. 2 *B* and *C*).

These results help explain the regional differences in β observed in Fig. 1. A simple regression shows that, when controlling for urbanization level, regional dummies converge toward the global average, with differences becoming either statistically insignificant or much smaller (Fig. 2*D*). This indicates that regional differences in β largely reflect each country’s stage in the urbanization process.

**Fig. 3.**
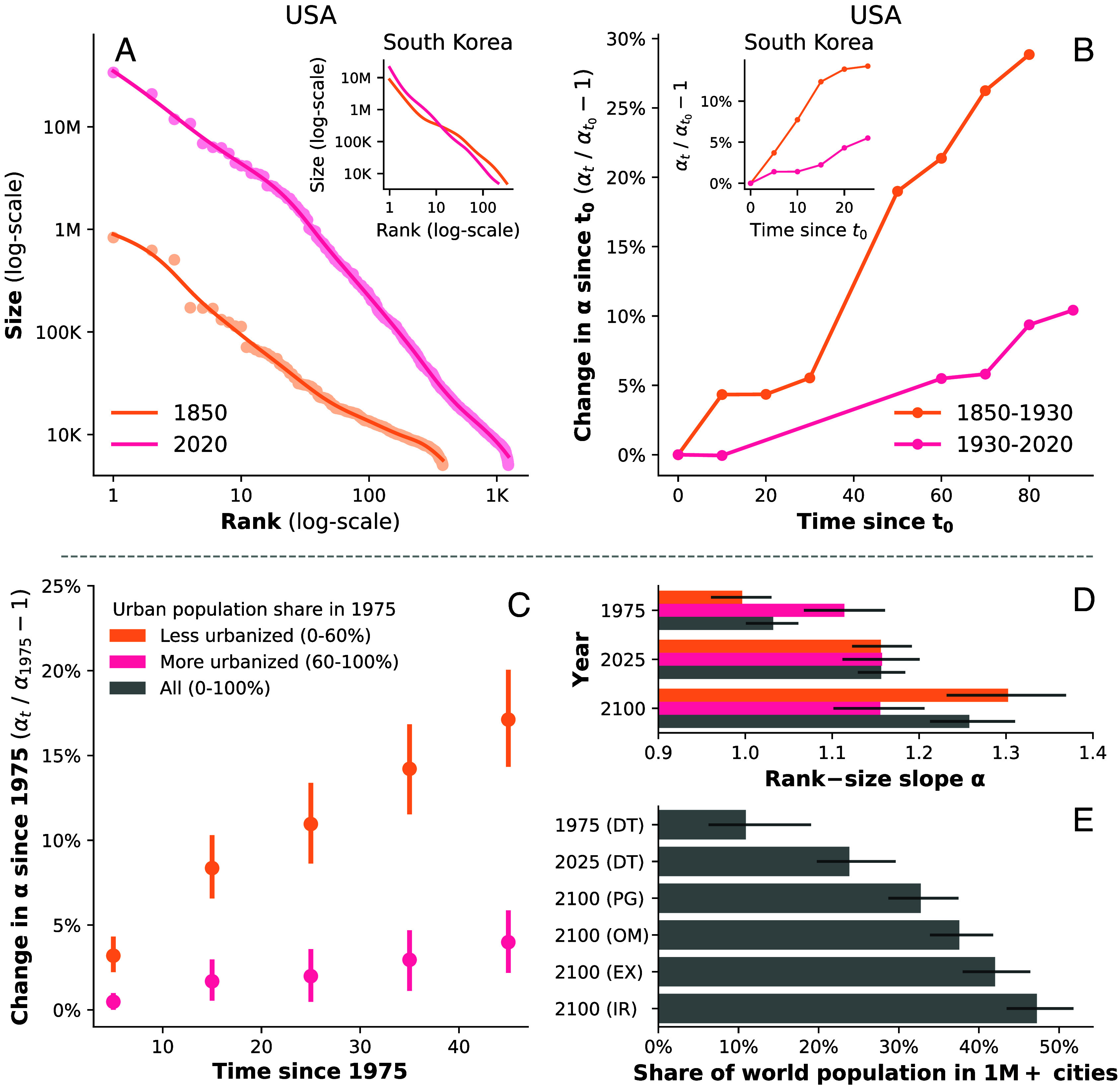
Historical trends and projections for national city size distributions. (*A*) Rank-size curves for the United States and South Korea. These curves are obtained by fitting penalized cubic B-splines (λ=1) to the relationship between city log-rank and city log-size in a fixed year. The US plot also displays a scatter of the underlying data points. (*B* and *C*) The rank-size slope α is estimated by averaging the local slope of the rank-size curve across ranks and then taking the absolute value (Section 3.3.2). Note that α is a spline-based analogue of the Zipf exponent. (*B*) Change in α for South Korea and the United States from a base year $t_{0}$ (South Korea $t_{0}=1975,\;2000$; USA $t_{0}=1850,\;1930$). (*C*) Change in average α across more/less urbanized countries since 1975 (binned scatter plot by year with bootstrapped CIs; n=1,000). (*D*) Average α across groups of countries in different years. 2100 values are projected using the technique in Section 3.4. (*E*) Share of the world population in 1M+ cities in 1975, 2025, and 2100. 2100 values are projected under four urban growth models, which differ in their estimates for the future trajectories of β: proportional growth (PG; β=0), increasing returns (IR; β=0.03), current trend extrapolation (EX; β equal to the country’s average between 1975 and 2025), and our model (OM; time-varying β according to the country’s urbanization). See Section 3.4.

The size–growth patterns observed in Fig. 2 change national city size distributions. We can quantify this change by looking at the distribution’s rank-size curve (Fig. 3*A*), or more precisely at the absolute value of its slope, α (exact definition of α in the caption of Fig. 3 *B* and *C*). If β is positive, large cities have a growth advantage, the left tail of the rank-size curve rises faster than the right one, the curve steepens, and α increases. Thus, if β declines with urbanization (as in Fig. 2), α should increase at a decelerating rate. Our data confirm this: α increased in both the United States and South Korea (Fig. 3*B*), and it did so faster early on. Further, since 1975, α grew 18% on average across less urbanized countries, against 4% across more urbanized ones (Fig. 3*C*).

With the mechanism in hand—β shapes α, and β weakens as countries urbanize—we move from description to prediction by projecting trajectories of national αs to 2100 (Section 3.4). Our projections suggest that growth in α will slow over the coming decades. Between 1975 and 2025, α grew by 2.3% per decade on average across the countries in our sample. Between 2025 and 2100, that same average is projected to grow at 0.9% per decade. Further, α will continue to grow faster in countries that were less urbanized in 1975. By 2100, their average α is projected to be 16% larger than that of the more urbanized group, while it was 12% smaller in 1975 (Fig. 3*D*).

Translating projected αs into population shares, we estimate that 38% of the world population will live in 1M+ cities by 2100 (Fig. 3*E*; Section 3.4). This projection sits below an extrapolation of current trends (42%; Fig. 3*E*) and above the proportional-growth benchmark (33%; Fig. 3*E*), consistent with a weakening growth advantage of large cities as urbanization progresses.

## 2. Discussion

Using a robust geographic definition of cities and a comprehensive database spanning several countries and historical periods, we show that urban growth follows a typical life cycle. Early in urbanization, large cities experience a strong growth advantage, stretching the city size distribution. As urbanization progresses, this growth advantage weakens and the distribution stabilizes. We use this model to forecast urban concentration at the end of the century. Relative to an extrapolation of 1975–2025 trends, our model projects 450 million fewer residents in 1M+ cities by 2100 (−4.4%); relative to proportional growth, 490 million more (+4.8%).

Our findings add nuance to prior work on the long-run concentration of population in large cities. Harmonized historical databases have documented this process across Europe since 1600 (44, 45) and across seven major world regions between 1960 and 2010 (46). We recover this concentration trend and reveal that it weakens as countries urbanize.

Our study has some limitations. First, our city definition is geographic rather than functional. It tracks clusters of built-up area or high population density, making it well suited for comparative analyses across countries and years. However, because it separates central cores from their surrounding suburbs, it may fail to fully capture the true gravitational pull of large cities. As a result, this definition might underestimate the growth advantage of large functional urban areas, especially in urbanized countries where advanced transport networks drive growth from city cores into surrounding suburbs and satellite settlements. Our supplementary analysis suggests that this is the case: Size–growth slopes estimated using geographic urban areas are typically flatter than those estimated using functional urban areas (*SI Appendix*, section 5.1). However, this underestimation is small relative to the overall trends, such that our findings do not substantially change when shifting the analysis to the level of functional urban areas. Accordingly, our projections should be read as conservative estimates of future concentration in functional urban areas.

Second, we use national borders to define urban systems. This definition is imperfect, especially in settings with strong cross-border integration. Our choice is dictated by both convenience and substance. With respect to convenience, countries are the territorial units that are most readily comparable in global analyses and that have the most widely available longitudinal information on their urban systems, both historically and for projections into the future. In more substantive terms, national borders strongly constrain the migration patterns, trade, institutions, and infrastructure that profoundly shape urban development (47). Developing a more principled approach to defining urban systems, perhaps through migration networks (48), is nevertheless a promising area for future research. To partially accommodate the limitations of relying on national borders to delineate urban systems, we provide a supplementary analysis at the level of UN M49 subregions (*SI Appendix*, section 4.1). Its findings align with our country-level ones, corroborating the declining growth advantage of large cities also at intermediate spatial scales.

Third, we focus on population, neglecting other aspects of city size, such as GDP, innovation, or employment. Population is the natural starting point for our analysis because it is the main measure of city size, it has been used for centuries, it is broadly comparable across countries, and it is central to the Gibrat, Zipf, and scaling literatures (6, 29, 36, 39, 49). However, extending our work to economic outcomes and measuring how scaling laws change over time is a valuable direction for future research (3, 10, 50). The IPUMS full-count census data used in this paper are an excellent starting point for such work (26, 28).

Fourth, our framework is phenomenological rather than causal. We document a robust empirical regularity and show how it may be used to improve projections. However, we do not isolate the causal mechanisms underpinning this regularity. Several mechanisms could cause the weakening of the growth advantage of large cities, including slowing rural-to-urban migration, reduced imbalances in migration flows (37, 48, 51), rising congestion and housing costs (23), diffusion of economic activity (5, 52), and the redistribution of metropolitan growth toward suburban peripheries (suburbanization) (53). In *SI Appendix*, section 5, we provide a brief discussion of the role of suburbanization, showing that its effect is real but insufficient to fully explain this phenomenon. Distinguishing the relative contributions of other causes is a promising area of future work.

Fifth, our projections are conditional on the absence of major external shocks—the standard *ceteris paribus* assumption. Because our model is not structural, we cannot explicitly integrate external shocks, such as geopolitical disruptions, climate-driven migration, pandemics, or technological revolutions. Any of these forces could, in principle, reshape urban trajectories in ways our model does not capture. For example, advances in urban planning and sanitation could reduce the diseconomies of scale that constrain large cities. Climate change could intensify migration toward hubs. That said, the urbanization pattern we document has proven remarkably robust. It has persisted through a profoundly volatile century, withstanding disruptions such as the automobile revolution, the internet, the rise and fall of geopolitical blocs, the construction of modern urban infrastructure, and the globalization of trade.

Despite the above limitations, our results have relevant implications when viewed through the lens of urban scaling theory (54). Because many urban outcomes scale nonlinearly with population, reallocating people across cities of different sizes changes aggregate outcomes (see *SI Appendix*, section 2.3 and refs. 6, 55, and 56). Our results suggest that as urbanization progresses, this reallocation becomes less skewed toward the largest cities and more evenly distributed across the urban system. The policy implications are mixed. On the one hand, if productivity scales superlinearly with city size, a slowdown in the growth of large cities implies a slowdown in reallocation-driven productivity growth (6, 9, 57). On the other hand, if emissions, urban heat islands, or other environmental burdens rise superlinearly with city size, the same slowdown may ease some environmental pressures (58, 59). Put simply, whether urban concentration or equalization is preferable depends on which of these trade-offs policymakers prioritize—a question that research can inform but not resolve.

## 3. Materials and Methods

### 3.1. Data.

This paper builds on several datasets, all of which are publicly available. Below we provide a high-level overview of the main datasets. In *Data, Materials, and Software Availability*, we document how to retrieve them.

#### 3.1.1. Population and urbanization.

The population projections come from the United Nations World Population Prospect 2024 (60), the History database of the Global Environment 2023 (61), and Gapminder (62, 63), with processing from Our World in Data (64). We use projections under the medium fertility scenario. The urbanization data come from Chen et al. (65). We use their World Bank-based annual projections under SSP2 (“middle of the road”), which extend the World Bank series to 2100. These data rely on national statistical definitions of “urban,” which vary across countries and differ from our morphological definition. We adopt these projections for three reasons. First, they are independently validated. Second, they are directly connected to future projections. Third, they provide more reliable estimates of the urban population share than GHSL grids, which are known to underestimate rural populations (25, 66). We therefore use GHSL grids only to compare urban areas, not to estimate the overall urban population share. Our results are robust to the use of a different definition of urban population share (*SI Appendix*, section 4.2).

#### 3.1.2. Country borders.

Borders are as of 2019, from the CShapes database (67).

#### 3.1.3. Global grids.

Our global cities dataset is based on the population (pop) and degree-of-urbanization (smod) grids from the Global Human Settlement Layer (GHSL) 2023 data package (25). These grids estimate population counts and urbanization levels for 1 km-by-1 km cells covering the whole planet by combining satellite imagery with census data.

#### 3.1.4. USA grids.

Our USA cities dataset is based on custom grids derived from census place population estimates from various data sources. For the 1990–2020 period, we use estimates from the National Historical Geographic Information System (IPUMS NHGIS) (27). For the 1850–1940 period, we reconstruct census place population estimates using the IPUMS USA full count census data (26) with geocoding from the Census Place Project (28). To do so, we match over 500 million individual census records to approximately 40,000 census places. We then aggregate these records to estimate the population of each census place. Because these historical data come from century-old handwritten documents, they contain numerous inconsistencies, such as census places disappearing and reappearing over time. We correct for these issues using several preprocessing steps that leverage individual migration data, as detailed in *SI Appendix*, section 1.1.

We use these clean census place population estimates to build population grids suitable for the City Clustering Algorithm. For each year t, we create a 1 km-by-1 km grid covering the continental US. Each grid cell $c_{j}$ receives an initial population ${\text{pop}}_{\text{init}}\left(c_{j}\right)$, given by the sum of the population of all census places whose geographic center falls within that cell. We then smooth this initial grid using a spatial convolution kernel: The final population of cell $c_{i}$ becomes a distance-weighted average of the initial population of neighboring cells $c_{j}$, with weights decreasing exponentially with distance $d_{c_{i}c_{j}}$:

[1] $$\begin{array}{c} \text{pop}\left(c_{i}\right)=\frac{1}{\sum_{j}e^{-\eta \cdot d_{c_{i}c_{j}}}}\sum_{j}e^{-\eta \cdot d_{c_{i}c_{j}}}\cdot {\text{pop}}_{\text{init}}\left(c_{j}\right)\;. \end{array}$$

The decay parameter η, which controls how quickly population decays around a census place, is set to η=0.2, similar to estimates in prior work (68).

### 3.2. Constructing Cities: The City Clustering Algorithm.

**Fig. 4.**
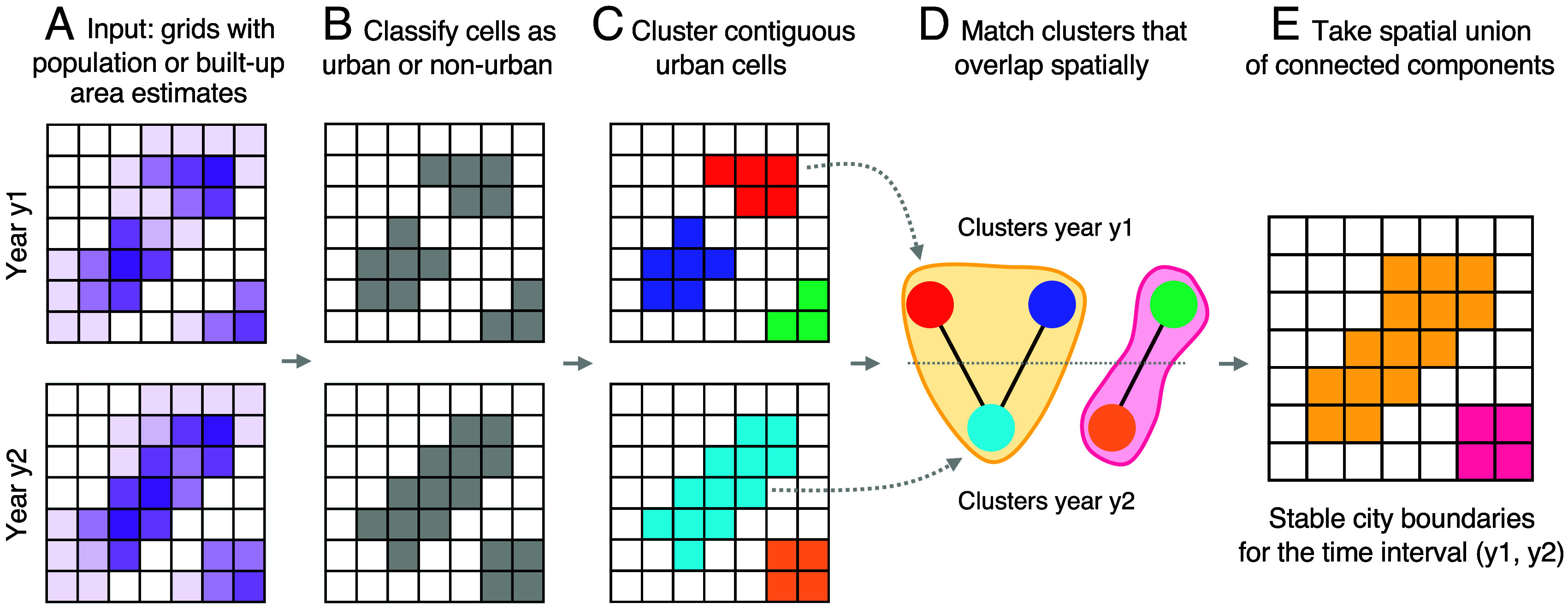
The City Clustering Algorithm (33) computes stable city boundaries for a time interval $\left(y_{1},y_{2}\right)$. This algorithm proceeds in five steps: (*A*) It takes two grids as input, one for each year, containing estimates of population or built-up area. (*B*) It classifies grid cells as either urban or nonurban using a threshold on these population/built-up area estimates. (*C*) It groups contiguous urban grid cells to form clusters. (*D*) It matches clusters across years when they overlap spatially, forming a bipartite graph. (*E*) It defines a city’s boundary as the spatial union of all clusters within a single connected component of the graph.

To construct cities from the United States and global grids, we use the City Clustering Algorithm (33). This algorithm identifies stable city boundaries between two years $t_{1}$ and $t_{2}$ based on the city’s geographic extent, allowing for robust measurement of population growth and comparable city definitions across countries and times. The algorithm, illustrated in Fig. 4, involves five steps:

1. **Classify urban cells**: For each year independently, we classify all grid cells as either “urban” or “nonurban” using population density or degree-of-urbanization thresholds.
2. **Form initial clusters**: For each year independently, we group contiguous *urban* cells into initial clusters using a flood fill algorithm (69).
3. **Match clusters over time**: For a pair of years $t_{1}\leq t_{2}$, we construct a bipartite graph G=(A,B), where nodes in part A are clusters in year $t_{1}$, and nodes in part B are clusters in year $t_{2}$. We connect clusters with an edge if and only if they overlap spatially.
4. **Define stable city boundaries**: We extract the connected components of the bipartite graph G that have at least one cluster in year $t_{1}$.^*^ Each component represents a single, evolving city that existed throughout the $\left(t_{1},t_{2}\right)$ period. The city’s boundary for the period is the spatial union of all clusters within the component (from both $t_{1}$ and $t_{2}$).
5. **Calculate population growth**: For each city, we calculate its population in years $t_{1}$ and $t_{2}$ by summing the population of all grid cells that fall within its boundary. The ratio of these two population values gives us the city’s growth rate over the period.

While the above algorithm largely follows the same approach as the original paper (33), we introduce some minor improvements and clarifications to its implementation.

First, using a bipartite graph to match clusters (Step 3) provides a more systematic and scalable approach than the manual specifications suggested in the original paper. It improves conceptual clarity, simplifies implementation, and naturally handles events like the merger or split of more than two clusters.

Second, our implementation is less ambiguous with respect to the “cell reclassification problem.” This problem occurs when cells flip their classification, moving from “nonurban” in year $t_{1}$ to “urban” in year $t_{2}$ (or vice versa). These flips do not require any dramatic transformation. As we use thresholds to determine “urban” and “nonurban” status, even small changes in population or built-up area can cause a flip. But if not treated carefully, flips may lead to biased growth estimates. For example, imagine a city where hundreds of grid cells flip from “nonurban” to “urban.” If we simply count the population in these newly “urban” cells as if they were entirely new additions to the city, we grossly overestimate its growth. Our solution (Steps 4 and 5) is to define a stable city boundary for the entire period and measure population changes only within that boundary. This isolates true population growth from the artifacts of reclassification. While this solution was adopted in the original CCA paper, the implementation is framed ambiguously and the problem is not mentioned.

Third, our implementation addresses a form of selection bias that we call “new cluster bias.” This bias arises if one includes urban clusters that appear in year $t_{2}$ but have no predecessors in year $t_{1}$. These “new” clusters represent only the fastest-growing locations among a larger pool of similar areas, many of which did not grow enough to become urban clusters. Including these successful outliers while ignoring the rest would upwardly bias the estimated growth rates for small cities. Our method avoids this by analyzing only components that contain at least one cluster from the start of the period (Step 4).

To produce the final datasets for our analysis, we apply the CCA to the United States and global grids with specific hyperparameters. We classified urban cells using distinct criteria for the United States and the global grids. For the US grids, we use a simple population threshold, classifying a cell as “urban” if it has more than 50 people. For the global grids, we use degree-of-urbanization estimates from the smod grid, classifying a cell as urban if it belongs to a “semidense urban cluster” or higher (cell value ≥22). Further, we applied a common city threshold to both datasets, filtering out clusters with fewer than 5,000 inhabitants. Finally, we excluded from the global cities dataset: i) Nepal and Myanmar due to data quality issues; ii) countries with fewer than 50 cities; iii) countries not present in the urbanization data (65). In *SI Appendix* sections 3.1 and 3.2, we discuss these hyperparameter choices in more detail and conduct sensitivity analyses confirming that our results are robust to reasonable variations in their values.

### 3.3. Analysis.

#### 3.3.1. Analytical framework.

We use a simple analytical framework to guide our analysis. While this does not constitute a causal theory of urban development, it provides a rigorous mathematical formalization that links size–growth slopes, rank-size slopes, and the population share in million-plus cities.

This analytical framework rests on two simplifying assumptions. First, a city’s size S(t) at time t is a power-law function of its (descending) rank R(t), meaning that the rank-size curve (x= log-rank; y=log-size) is a straight line with slope $a_{t}$:^†^

[2] $$\begin{array}{c} S\left(t\right)\propto R{\left(t\right)}^{-a_{t}} \end{array}$$

Second, a city’s growth rate between t and t+10 is a power-law function of its size S(t), meaning that the size–growth curve (x=log-size; y=log-growth) is a straight line with slope $b_{t}$:

[3] $$\begin{array}{c} g\left(t,S\left(t\right)\right)=\frac{S(t+10)}{S(t)}\propto S{\left(t\right)}^{b_{t}}. \end{array}$$

Under these assumptions, $a_{t}$ and $b_{t}$ are related by a simple equation:

[4] $$\begin{array}{c} a_{t+10}=a_{t}\cdot \left(1+b_{t}\right). \end{array}$$

For $t_{1}>t_{0}$, this equation generalizes to:

[5] $$\begin{array}{c} a_{t_{1}}=\;\exp(\log\left(a_{t_{0}}\right)+\sum_{s\in t_{0},t_{0}+10,\cdots ,t_{1}-10}\log\left(1+b_{s}\right)). \end{array}$$

Furthermore, $a_{t}$ is linked to the share of the *urban* population living in 1M+ cities, $m_{t}$. In fact, the probability of observing a city larger than size x in year t is given by $P\left(S\left(t\right)>x\right)\propto x^{-1/a_{t}}$, so:

[6] $$\begin{array}{cc} m_{t} & =\int_{z}^{x_{t,\max}}P\left(S\left(t\right)>x\right)dx\;/\;\int_{x_{t,\min}}^{x_{t,\max}}P\left(S\left(t\right)>x\right)dx \end{array}$$

[7] $$\begin{array}{cc} & =\frac{x_{t,\max}^{1-1/a_{t}}-z^{1-1/a_{t}}}{x_{t,\max}^{1-1/a_{t}}-x_{t,\min}^{1-1/a_{t}}}. \end{array}$$

Here, $x_{t,\max}$ and $x_{t,\min}$ are upper and lower bounds on the size of a country’s cities, and $z=10^{6}=\text{1M}$.

In sum, this analytical framework provides simple closed-form equations relating the growth advantage of large cities (b) to the concentration of population within them (a, m).

#### 3.3.2. Empirical measurement.

The above framework highlights a and b as the core parameters governing the evolution of urban systems. We estimate these parameters as follows:

(a) The rank-size slope α is our empirical estimate for the parameter a. To obtain α we first estimate the rank-size curve h by fitting a penalized cubic B-spline (with penalty λ=1) to city log-rank vs. city log-size data points:
  [8] $$\begin{array}{c} \log_{10}(S\left(t\right))=h(\log_{10}\left(R\left(t\right)\right)). \end{array}$$
  Then we take the absolute value of the mean derivative of h over the log-rank spectrum ($r=\;\log_{10}\left(R\right)$):
  [9] $$\begin{array}{c} \alpha =\frac{1}{r_{\max}-r_{\min}}|\int_{r_{\min}}^{r_{\max}}h^{\prime }\left(r\right)dr|=|\frac{h\left(r_{\max}\right)-h\left(r_{\min}\right)}{r_{\max}-r_{\min}}|. \end{array}$$
(b) The size–growth slope β is our empirical estimate for the parameter b. As above, we obtain β by first estimating the size–growth curve f, a penalized cubic B-spline (with penalty λ=100) fit to the city log-size vs. city log-growth data points:
  [10] $$\begin{array}{c} {\text{log}}_{10}(g\left(t,S\left(t\right)\right))=f({\text{log}}_{10}\left(S\left(t\right)\right)). \end{array}$$
  Then we take the mean derivative of f over the observed log-size spectrum ($s=\;{\text{log}}_{10}\left(S\right)$):
  [11] $$\begin{array}{c} \beta =\frac{1}{s_{\max}-s_{\min}}\int_{s_{\min}}^{s_{\max}}f^{\prime }\left(s\right)ds=\frac{f\left(s_{\max}\right)-f\left(s_{\min}\right)}{s_{\max}-s_{\min}}. \end{array}$$

We also estimate size–growth curves for groups of countries (such as Europe or less urbanized countries in 1975). The procedure to estimate a group-level size–growth curve $f_{g}$ comprises three steps. First, we normalize each city’s growth rate $g\left(t,S_{\mathrm{ic}}\left(t\right)\right)=S_{\mathrm{ic}}\left(t+10\right)/S_{\mathrm{ic}}\left(t\right)$ by the average city growth rate of its country c:

[12] $$\begin{array}{c} g_{c}\left(t\right)=\sum_{i\in c}S_{\mathrm{ic}}\left(t+10\right)/\sum_{i\in c}S_{\mathrm{ic}}\left(t\right)=\sum_{i}\frac{S_{\mathrm{ic}}\left(t\right)}{\sum_{i\in c}S_{\mathrm{ic}}\left(t\right)}\cdot g\left(t,S_{\mathrm{ic}}\left(t\right)\right). \end{array}$$

Second, we pool the normalized growth rates from all countries in the group and fit a single penalized cubic B-spline ${\overset{¯}{f}}_{g}$ (with λ=100):

[13] $$\begin{array}{c} {\text{log}}_{10}(\frac{g(t,S_{\mathrm{ic}}\left(t\right))}{g_{c}\left(t\right)})={\overset{¯}{f}}_{g}({\text{log}}_{10}\left(S_{\mathrm{ic}}\left(t\right)\right)). \end{array}$$

Third, we recenter the function ${\overset{¯}{f}}_{g}$ by the group’s average growth rate:

[14] $$\begin{array}{c} f_{g}={\overset{¯}{f}}_{g}+\frac{1}{\left|g\right|}\sum_{c\in g}\log_{10}\left(g_{c}\left(t\right)\right). \end{array}$$

Other authors use ordinary least squares (OLS) regression to estimate the parameters a and b. Our approach has two advantages over the OLS approach. First, the resulting parameter estimates are more robust to arbitrary design choices, such as the lower threshold for city size. Second, the estimates align more closely with the equations derived from the analytical framework, even when the data deviate from the framework’s ideal assumptions (*SI Appendix*, Figs. S4 and S5). These advantages can be explained by how each approach calculates average slopes. Our approach calculates the average slope of a given curve by putting equal weights on each part of the x-range. The OLS approach, in contrast, weights parts of the x-range by the density of data that are within them. Because small cities dominate city population data, the OLS slope is disproportionately influenced by the small-city end of the curve. This makes the OLS slope susceptible to variation in the city size lower threshold and less consistent with our theoretical equations. In *SI Appendix*, section 2.1, we compare spline-based and OLS-based parameter estimates in greater detail.

### 3.4. Projections.

The core idea of our projection method is to take the closed-form equations from Section 3.3.1 and populate them with our spline-based measurements from Section 3.3.2. The logic is that the equations capture the form of the relationship between parameters, while our spline-based measurements provide the most accurate values for these parameters (*SI Appendix*, section 2.2).

We start by projecting β. We consider four scenarios:

- Proportional growth: We set $\beta_{\mathrm{tc}}=0$ for all t≥2020 and all countries c.
- Increasing returns: We set $\beta_{\mathrm{tc}}=0.03$ for all t≥2020 and all countries c.
- Current trend extrapolation: We set $\beta_{\mathrm{tc}}={\overset{¯}{\beta }}_{c}$ for all t≥2020 and all countries c. Here, ${\overset{¯}{\beta }}_{c}$ is the average β for country c over 1975–2025 (as mapped in Fig. 1*C*).
- Our model: We regress β on the urban population share u to obtain ρ=−0.049 (Table 2). Then, for t≥2020, we set:
  [15] $$\begin{array}{c} \beta_{\mathrm{tc}}={\overset{¯}{\beta }}_{c}+\rho \cdot \left(u_{\mathrm{ct}}-{\overset{¯}{u}}_{c}\right). \end{array}$$
  Here, $u_{\mathrm{ct}}$ is the projected urban population share for country c from ref. 65 (Section 3.1) and ${\overset{¯}{u}}_{c}$ is the average urban population share for country c over 1975–2025.

We project α for t≥2030 by plugging the projected βs into an empirical version of Eq. **5**:

[16] $$\begin{array}{c} \alpha_{t}=\;\text{exp}(\text{log}\left(\alpha_{2020}\right)+\sum_{s\in 2020,\cdots ,t-10}\log\left(1+\beta_{s}\right)). \end{array}$$

We project the share of *total* population living in 1M+ cities using a two-step approach.

1. First, we project the share of *urban* population in 1M+ cities using an empirical version of Eq. **6**:
  [17] $$\begin{array}{c} m_{t}=\frac{x_{t,\max}^{1-1/\alpha_{t}}-z^{1-1/\alpha_{t}}}{x_{t,\max}^{1-1/\alpha_{t}}-x_{t,\min}^{1-1/\alpha_{t}}}. \end{array}$$
  The key challenge in evaluating the right-hand side of this equation is to set plausible values for $x_{t,\min}$ and $x_{t,\max}$, the lower and upper bounds on the sizes of a country’s cities. For $x_{t,\min}$, we use the city size lower bound used throughout the paper, $x_{t,\min}=5,000$. For $x_{t,\max}$, the choice is less straightforward because there is no clear upper bound for the size of a country’s cities. We assume that $x_{t,\max}$ is proportional to the total urban population of a country $U_{t}$, i.e., $x_{t,\max}=\omega \cdot U_{t}$ where ω is a tunable parameter. We then calibrate ω using historical data. For each country, we select ω∈(0.1,2) to minimize the average deviation between the shares $m_{t}$ calculated using Eq. **17** and the shares ${\hat{m}}_{t}$ observed directly in the data. Aggregated at the regional level, the calibrated estimates $m_{t}$ closely match the observed data ${\hat{m}}_{t}$ (mean absolute error $|m_{t}-{\hat{m}}_{t}|$ below 0.01; see *SI Appendix*, Fig. S6).
2. Second, we multiply the projected shares $m_{t}$ by the projected urban population share $u_{t}$ to obtain the share of *total* population living in 1M+ cities.

## Supplementary Material

Appendix 01 (PDF)

## Data Availability

All data and source code underlying this paper are publicly available. To enable exact re-execution, we provide a fully automated, end-to-end pipeline that reproduces the entire analysis with a single command. The pipeline is available on GitHub https://github.com/ethz-coss/global-city-growth (70) and archived on Zenodo at the time of publication https://doi.org/10.5281/zenodo.17339402 (71). It handles ~300 GB of data spanning heterogeneous formats like raster, vector/shapefile, CSV, and Parquet. It executes 100+tasks in a directed acyclic graph and produces 200+ output tables persisted in PostgreSQL (primary store) and processed with DuckDB (for fast processing of large tables). It is containerized with Docker. On a MacBook Pro (Apple M2, 32 GB RAM), a full run completes within ~5 h. We deposited all redistributable source data at https://doi.org/10.5281/zenodo.17240843 (72). Some datasets (IPUMS and NHGIS) are public but not redistributable. The pipeline retrieves them via the IPUMS API. Running the pipeline requires a personal IPUMS API key (see https://developer.ipums.org/docs/v2/apiprogram/) (73). A full database dump of all aggregate tables resulting from a pipeline run is available at https://doi.org/10.5281/zenodo.17338967 (74). The key output datasets—city boundaries and populations for the USA (1850–2020) and the world (1975–2025) and projections of population shares in 1M+ cities by country—are available at DOI: 10.5281/zenodo.17315337 (75).

## Footnotes

^*^We do so to avoid “new cluster bias” (see below).

^†^Note that with this definition the complementary cumulative density function (CCDF) of the city size distribution is $P\left(S>x\right)\propto x^{-1/a_{t}}$. Other authors define the exponent with its reciprocal $\zeta_{t}=1/a_{t}$ so that the CCDF is $P\left(S>x\right)\propto x^{-\zeta_{t}}$.
