# Supplementary material for: Large cities lose their growth advantage as countries urbanize
Source: Proc Natl Acad Sci U S A. 2026 Jun 25;123(26):e2529430123. doi: 10.1073/pnas.2529430123 (PMC13321366; doi:10.1073/pnas.2529430123)
Supplement: Supplementary file 1 — Appendix 01 (PDF) [file pnas.2529430123.sapp.pdf]

Supporting Information for

Large cities lose their growth advantage as countries urbanize

Andrea Musso, Diego Rybski, Dirk Helbing, and Frank Neffke

Andrea Musso.
E-mail: andrea.musso@gess.ethz.ch

This PDF file includes:

Figs. S1 to S10
Tables S1 to S5
SI References

1. Data and definitions

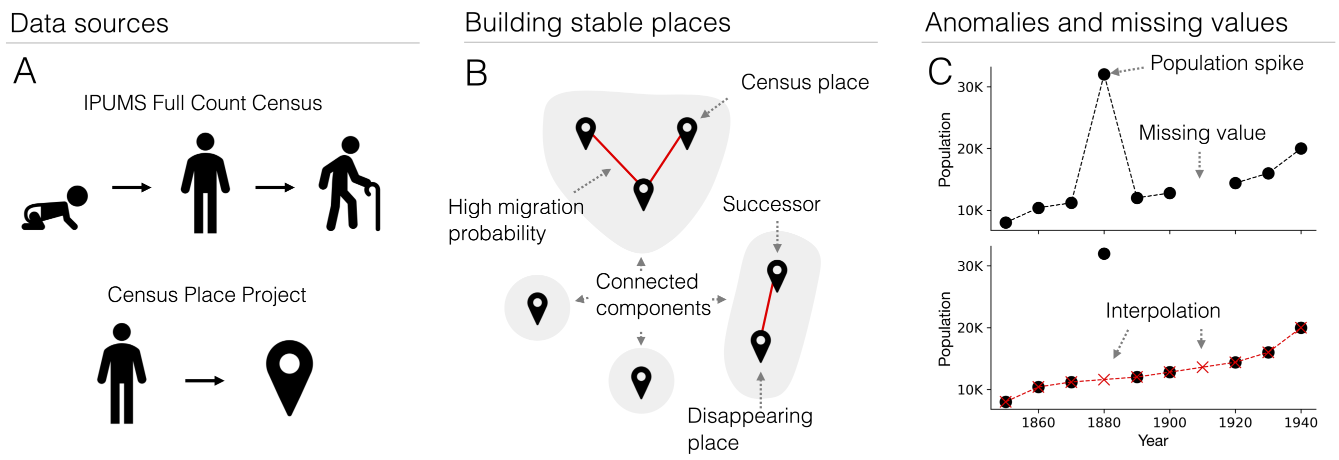

Fig. S1. Harmonization of historical US census data. **(A)** Our census place population estimates build on two data sources: the Census Place Project and the IPUMS full count census. The Census Place Project maps individuals to historical census places, allowing us to infer their population. The IPUMS full count census links individuals across census years, allowing us to compute migration flows between census places. **(B)** The historical population data contains significant noise, such as census places that disappear or exhibit implausible population spikes. We resolve these issues by constructing a network based on migration flows, where the connected components of the network define geographically and temporally stable places. **(C)** We use linear interpolation to fix anomalies and impute missing values in the population time series of our stable census places.

A. Harmonization of historical US census data. Our US cities data between 1850 and 1940 comes from two primary data sources: the IPUMS full count census and the Census Place Project (Supp. Fig. S1A).

The IPUMS full count census is the result of a massive digitization project that transformed all US censuses into machine-readable format. These machine-readable censuses contain a wealth of socio-demographic information about every single person who lived on US soil. The key information used here is the census linking key: a unique identifier that tracks individuals across different censuses, making it possible to trace their movements over time.

The Census Place Project complements the IPUMS full count census by providing more granular geographic information about the location of individuals. Using a complex matching procedure on the strings of a digitized version of the original handwritten censuses, the authors map each individual in the census to one of almost 70,000 *historical census places*.

Both datasets draw on historical USA decennial census records, which have been lawfully released to the public. The version used in this paper is a public-use, coded dataset that omits direct identifiers such as names and street addresses. Our analysis relies on these lawfully released, de-identified records and, since we use them only to construct aggregate place-level measures, complies with applicable data protection and privacy laws.

Our pipeline uses the Census Place Project to estimate the population of historical census places, and the linking from the IPUMS full count census to denoise these estimates. It features four steps.

In the first step, we standardize the definition of census place across our data. The historical data (1850-1940) uses "historical census place" as its base unit, whereas the modern data (1990-2020) uses "NHGIS census place". We match the 70,000 historical census places to 34,000 NHGIS census places by assigning each historical place to its nearest NHGIS neighbor and adopt the NHGIS census place as our base unit.

In the second step, we use the above matching to estimate the population of NHGIS census places during the historical period (1850-1940). These estimates have two key issues: (i) there are a non-negligible number of census places (around 17% of the total) that disappear: they have a non-zero population in some year t_e and zero population in years $t > t_e$; (ii) there are population spikes: given three consecutive observation years t_1, t_2 and t_3, the population of the census place doubles between years t_1 and t_2 and then halves between t_2 and t_3. This noise is not surprising. The data come from handwritten documents that are over one hundred years old.

In the third step, we address this noise by re-allocating population across census places. To support our re-allocation decisions, we estimate migration flows between places using the census linking. This unique identifier allows us to follow the movement of individuals across different censuses and hence estimate the probability of migration between places. We use these migration probabilities to match disappearing places with successors. For each disappearing place, we identify its successor as the location within a 50 km radius that has the highest probability of receiving its migrants. If migration data is unavailable, we simply choose the nearest place.

This process results in a network where nodes are census places and edges link each disappearing place to its assigned successor (Fig. S1B). We enrich this network by adding an edge between two places if the migration probability between them is "high", where high means that two places within 50 km distance have a migration probability exceeding 0.5 (50% of the people living in one place moved to the other between two observations).

If two places in the network are linked by an edge, we consider them the same place. So, the connected components of the network identify unique places. We assign each connected component a unique identifier using the NHGIS ID of the member of the component with the highest total population. We then estimate the population of each connected component by summing the populations of its members. This results in a dataset of 30,000 census places that are stable throughout our historical period.

In the fourth and final step, we clean the population time series of our stable census places. First, we apply heuristic rules to flag implausible data points, such as extreme population spikes (Fig. S1C). Then we use linear interpolation to impute these flagged data points and existing missing values. The result is a harmonized panel dataset covering the populations of 30,000 census places from 1850 to 1940. The combination of this dataset and the much cleaner modern version (1990-2020) powers our longitudinal analysis of the US.

B. A visual comparison of administrative, functional, and geographic city definitions. Broadly speaking, city definitions fall into three categories: administrative, functional, and geographic. In Figure S2, we illustrate these different definitions for four large cities: Paris, New York, London, and Rio de Janeiro.

Administrative definitions are based on political boundaries. Their main strength is that they are widely available. From small towns to sprawling megacities, most settlements have well-defined political boundaries. However, these boundaries are rigid or slow to change, leading to a distorted picture of a city's actual growth. For example, all four political cities (orange) in Figure S2 are far smaller than their urban areas (red), indicating that these cities have spilled over their political boundaries.

Functional definitions (like the widely used Metropolitan Statistical Area (MSA)) are based on commuting patterns. These definitions often encompass the urban area itself and a wider region surrounding it from where people commute for work (see blue areas in Figure S2). These areas provide the most accurate city definition from an economic perspective because they capture the gravitational pull of the city beyond its geographical boundaries. The core weakness of functional definitions lies in their coverage and comparability. Functional urban areas are typically constructed by hand on a case-by-case basis, limiting their availability to large-ish cities. Additionally, criteria for defining these areas change across countries and over time, limiting comparability.

Geographic definitions are based on built-up area or population density thresholds. The city boundaries issued from these definitions tend to coincide with those of the "urban area", as defined by different statistical agencies. Intuitively, they coincide with the agglomeration of built-up area (Fig. S3), and so their boundaries lie somewhere in the middle between the broader functional urban area and the smaller administrative area (Fig. S2). Their disadvantage is that they fail to capture the gravitational pull of the city beyond its built-up area. Their advantages are that they adapt naturally as cities expand, and they are both widely available and highly comparable. In fact, advances in satellite imagery and algorithmic processing make it possible to track an unprecedented range of settlements over long time windows, all while keeping similar city definitions. While no definition is perfect, our view is that geographic definitions offer the best balance between coverage, comparability, and adaptation for studying city growth on a large spatial and temporal scale.

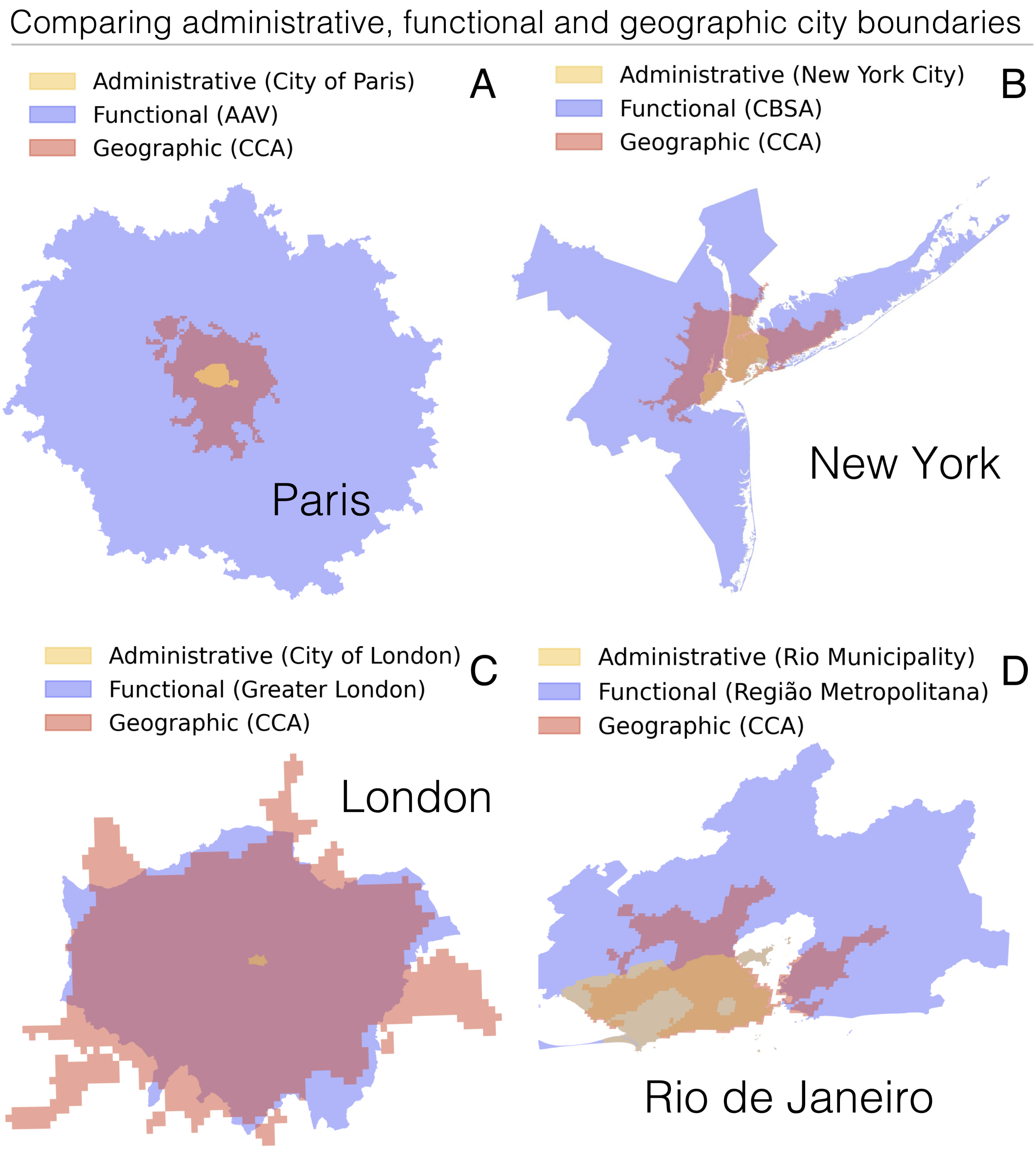

Fig. S2. A comparison of administrative, functional, and geographic city definitions for (A) Paris, (B) New York, (C) London, and (D) Rio de Janeiro. The geographic definition (red) is the one used in this study, derived by applying the City Clustering Algorithm (CCA) to the grids from the Global Human Settlement Layer. The administrative (orange) and functional (blue) boundaries are based on the following official definitions: **(A)** Paris: The administrative area is the City of Paris (the 20 arrondissements); the functional area is the Aire d'attraction d'une ville (AAV), which is based on commuting patterns. **(B)** New York: The administrative area is New York City (the five boroughs); the functional area is the Core-Based Statistical Area (CBSA), which is based on economic and commuting ties. **(C)** London: The administrative boundary is the historic City of London; the functional boundary is the Greater London area, an administrative region composed of the City of London and 32 surrounding boroughs, which largely coincides with the area defined by commuting patterns. **(D)** Rio de Janeiro: The administrative boundary is the Rio Municipality; the functional boundary is the Região Metropolitana, an area of socio-economically integrated municipalities defined by state law for the joint planning of public services.

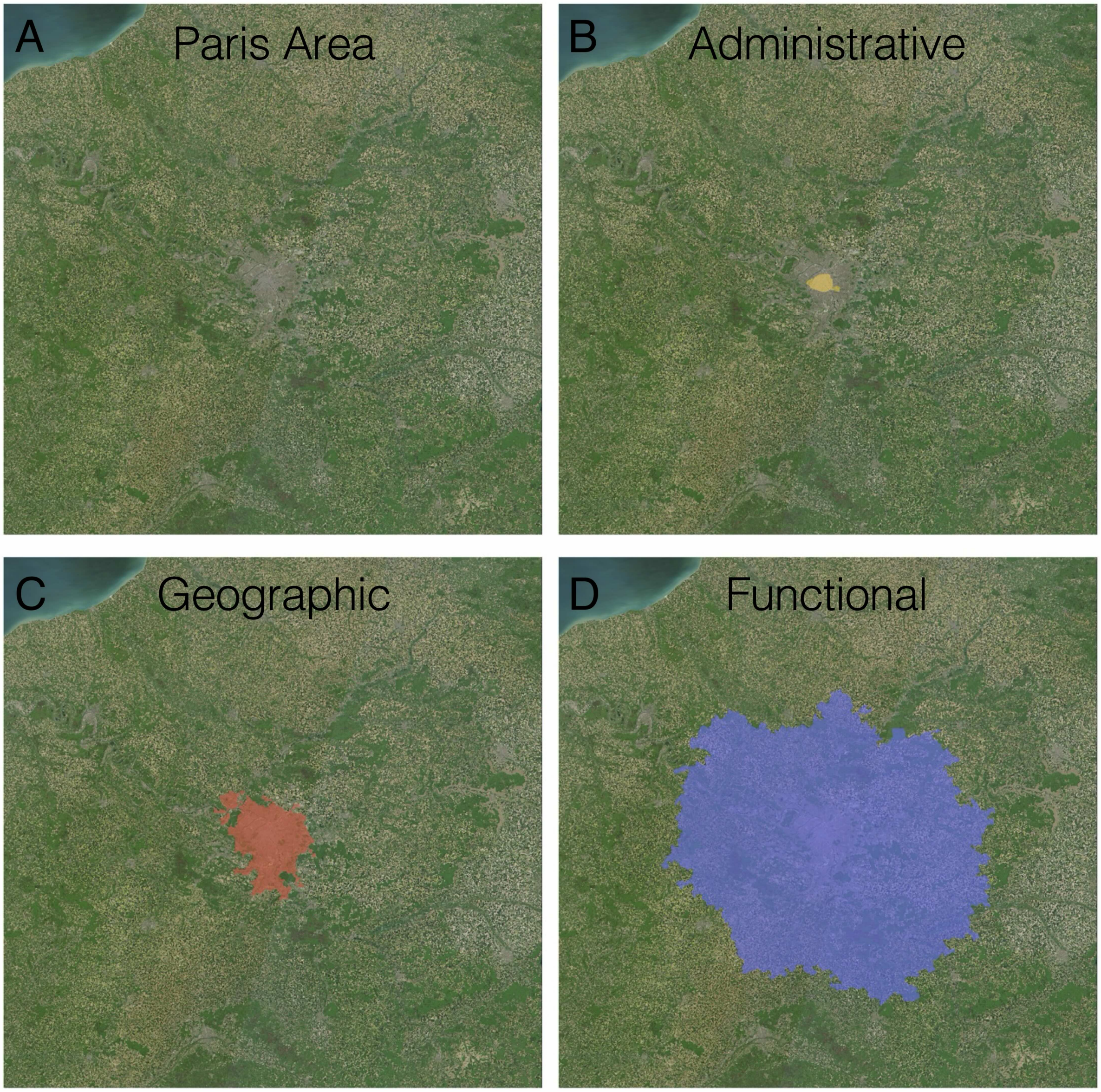

Fig. S3. Three distinct definitions of “Paris” against a satellite image of northern France. The administrative definition (orange) covers just the core of the continuous built-up area. The geographic definition (red) roughly coincides with this built-up area. The functional definition (blue) extends significantly further, encompassing a broad region of northern France that includes numerous surrounding rural areas with close economic or social connections to the urban center.

2. Methodology

A. Spline-based vs OLS-based parameter estimates. In this section, we explain why we prefer using spline-based methods over traditional linear models for measuring the parameters a and b of our theoretical model. Generally, we want our empirical estimates α, β for a, b to satisfy three desirable properties:

(i) α and β coincide with a and b when the model assumptions hold exactly, i.e., when the rank-size and size-growth curves are straight lines in log-log space.

(ii) α and β are robust to arbitrary empirical design choices, such as the city size lower threshold.

(iii) α and β are consistent with the equations derived from the theoretical model (e.g., $\alpha_{t+10} = \alpha_t \cdot (1 + \beta_t)$) even when the empirical data deviate from the perfect linearity assumed by the model.

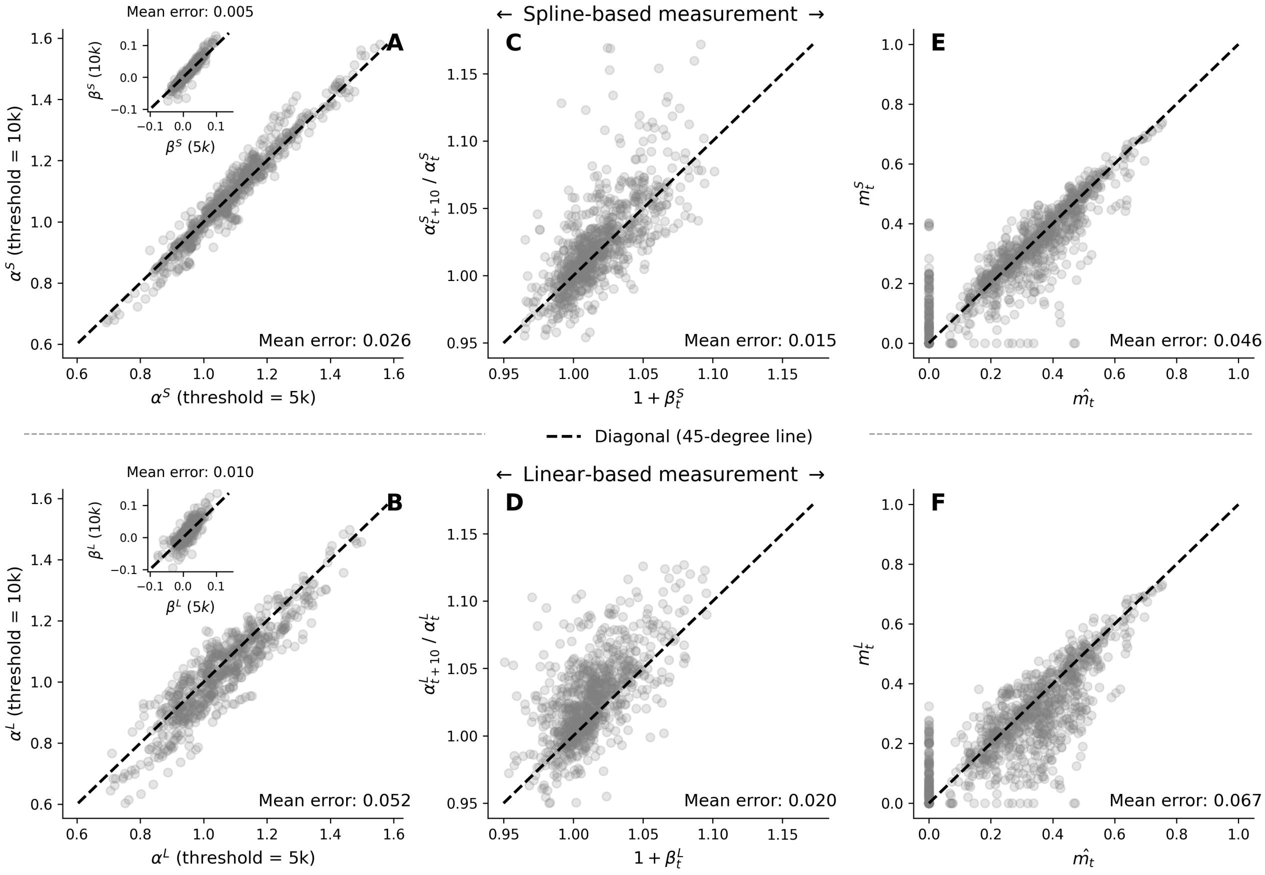

Fig. S4. Comparison of spline-based and OLS-based parameter estimates. **(A-B)** Scatterplots showing α and β estimated with a city size threshold at 5,000 vs. 10,000 people for (A) spline-based and (B) OLS-based estimates. **(C-D)** Scatterplots of α_{t+10}/α_t against $1 + \beta_t$ for (C) spline-based and (D) OLS-based estimates. For visual clarity, we omit the top and bottom 1% of data points so that extreme values do not compress the axes and obscure the central pattern. **(E-F)** Scatterplots of model shares m_t vs. observed shares $\hat{m}_t$ for (E) spline-based and (F) OLS-based estimates.

Our spline-based α^S, β^S satisfy these three properties more closely than OLS-based α^L, β^L. Property (i) is clearly satisfied by both. For property (ii), Figure S4A-B shows that α^S, β^S are more robust to a change in city size threshold from 5,000 to 10,000 people. For property (iii), Figure S4C-F shows that our two key equations hold more closely with spline-based estimates. For the equation relating a and b, points align more clearly on the 45-degree line and have a 25% lower mean absolute error $|\alpha_{t+10}/\alpha_t - (1 + \beta_t)|$ (Fig. S4C-D). For the equation relating a and m (the share of urban population living in 1M+), the spline-based estimate m_t^S fits the empirical data $\hat{m}_t$ better, reducing the mean absolute error by approximately 30% compared to the OLS-based estimate m_t^L (Fig. S4E-F).

We speculate that the greater robustness of our spline-based estimates has to do with weighting. Our spline-based slopes summarize a curve by giving *equal weight* to each part of the x-range. In contrast, the OLS slope summarizes a curve by giving different weights to different parts of the range, based on the density of data points within them. Put differently, the OLS slope is a *data-weighted* average of many local slopes that places more weight where observations are more dense. In city population data, small cities are far more numerous than large ones, so the OLS slope is dominated by outcomes in the small-city end of the curve. As a result, the OLS slope summarizes the sample composition more than the shape of the relationship. This

makes the OLS slope less robust to changes in the city size lower threshold, because small cities are weighted so heavily. It also makes OLS slopes less consistent with our theoretical equations, because these describe a curve's geometry independent of how densely observations are distributed along it.

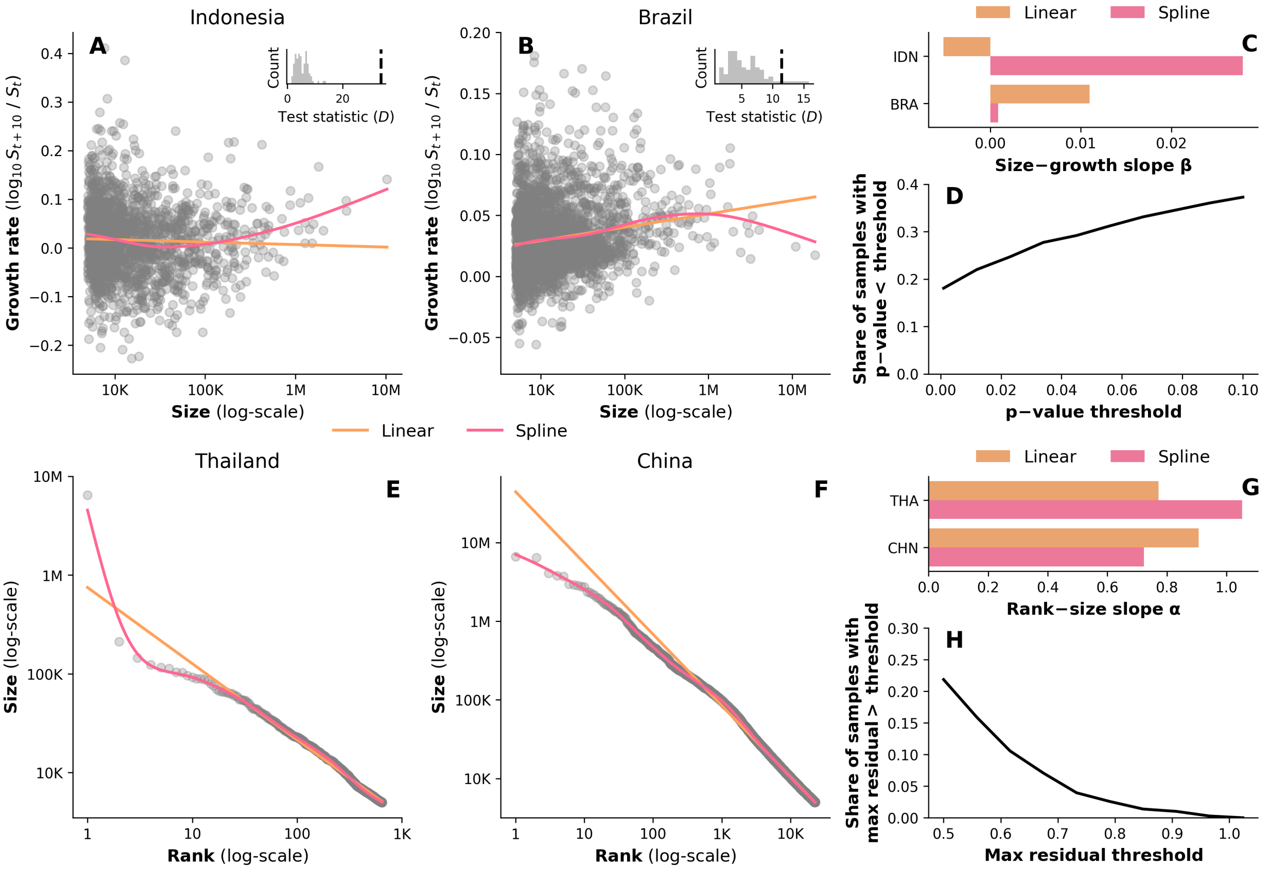

Fig. S5. Examples of OLS slopes poorly fitting size-growth and rank-size curves. **(A-B)** Size-growth curves for Indonesia and Brazil (pink = penalized B-spline fit with $\lambda = 100$, orange = OLS fit, grey = data points). Insets: the distribution (grey) and test statistic (dashed black line) of the log-likelihood ratio test described in the text. **(C)** Slope of the size-growth relationship (β) from an OLS-based/spline-based estimate. **(D)** Results of the log-likelihood ratio test by p-value threshold: approximately 30% of country-years reject linearity at $p < 0.05$. **(E-F)** Rank-size curves for Thailand and China (pink = penalized B-spline fit with $\lambda = 1$, orange = OLS fit, grey = data points). **(G)** Slope of the rank-size relationship (α) from an OLS-based/spline-based estimate. **(H)** Share of country-years with maximum-residual above threshold: more than 20% of samples have a maximum residual greater than 0.5.

Figure S5A-B shows examples in which OLS slopes provide poor estimates for the shape of the size-growth curve. The figure highlights a U-shaped curve in Indonesia, meaning that small and large cities grow faster than middle-sized ones. It also highlights an inverse-U shaped curve in Brazil, meaning that small and large cities grow slower than middle-sized ones. When we fit these patterns with an OLS, the slope only reflects what is happening at the left end of the data (small to medium size). As a result, the OLS slope provides a poor summary of the full curve. In Indonesia, large cities are growing much faster than the rest, but the OLS slope is negative. Conversely, in Brazil, large cities are growing slower, but the OLS slope is positive (Fig. S5C).

Bent curves are quite common, so this problem shows up in a non-negligible number of cases. We can see this through a simple log-likelihood ratio test. Our null hypothesis H_0 is that the relationship between log-size and log-growth in a given country and year is linear. Our alternative hypothesis H_1 is that it is not linear. Our test statistic is the log-likelihood ratio, $D = 2 \cdot (l_{\text{spline}} - l_{\text{linear}})$, which measures the improvement in fit offered by the spline model with respect to a linear one. If D is large, we know that the spline model greatly improves the fit, suggesting that the data present some important non-linearities, and hence that the linear model is likely misspecified.

To determine what "large" means for our test statistic D, we estimate it under the null hypothesis (linearity) using a wild bootstrap procedure. This procedure involves the following steps:

1. We fit a linear model to the original data using OLS regression.

2. We generate a synthetic dependent variable by adding the linear model's predicted values $\hat{y}$ to its residuals $y - \hat{y}$ multiplied by a random sign w:

$$y_{\text{boot}} = \hat{y} + w \cdot (y - \hat{y}) \qquad w = \text{random weights, -1 or +1 with probability 0.5} \,. \tag{1}$$

3. We estimate the linear and spline model with this synthetic dependent variable to obtain the log-likelihoods $l_{\text{linear}}^{\text{boot}}$ and $l_{\text{spline}}^{\text{boot}}$ and the bootstrapped test statistic $D_{\text{boot}} = 2 \cdot (l_{\text{spline}}^{\text{boot}} - l_{\text{linear}}^{\text{boot}})$.

4. We repeat steps 2 and 3 for $N = 1000$ iterations to obtain a distribution of plausible values of our test statistic under the null-hypothesis.

We define our test p-value by $p = P(D \leq D_{\text{boot}})$, i.e., the probability that the true test statistic D is smaller than the test statistics generated under the null hypothesis. If $p < 0.05$ we know that our true statistic is larger than 95% of the values generated under the null hypothesis — D is "large" — and hence that the data presents non-linearities (Fig. S5D). The insets of Figure S5A-B show the observed test statistic and the bootstrapped distribution for Indonesia and Brazil.

Figure S5E-F shows examples in which OLS slopes provide poor estimates for the shape of the rank-size curves. We observe a common example of non-linearity: urban primacy. Bangkok — Thailand's largest city — is over 30 times larger than the next city and home to more than 50% of Thailand's urban population. So, as we move from rank 1 to rank 2 in the Thai urban system, the slope of the rank-size curve declines far faster than when moving between higher ranks. The OLS slope fails to capture this pattern. Skewed by the many small cities, the slope largely ignores the outsized Bangkok. The result is a poor summary of Thailand's rank-size curve. Intuitively, Thailand has a very steep rank-size curve — the majority of people live in the biggest city — but the OLS slope suggests the opposite (Figure S5G).

Thailand is one of the most extreme cases, but non-linear rank-size curves are fairly common (e.g., China in Fig. S5F). To see this, we fit an OLS regression to log-rank vs. log-size data points, and then consider the maximum residual of this linear fit. The larger this maximum residual, the worse the curve fits the data. We observe that maximum residuals vary between 0.05 (very small) and 1.1 (large), with about 20% of the sample having a maximum residual above 0.5 (Fig. S5H).

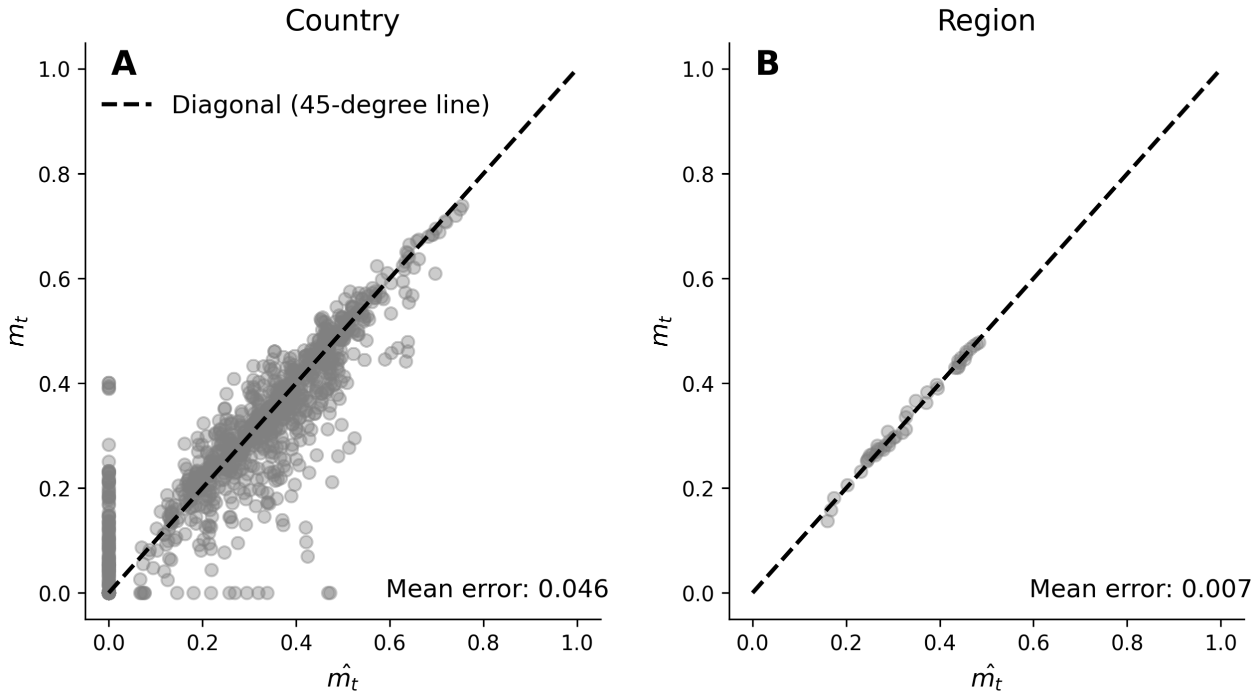

Fig. S6. Relationship between the modeled share of the urban population living in cities with at least one million people, m_t, and the observed share $\hat{m}_t$. The scatterplots show m_t vs. $\hat{m}_t$ at the **(A)** country level and **(B)** regional level. In both panels, points cluster along the diagonal (45-degree) line, indicating a good fit.

B. Projections. In this section, we explain how we calibrate the model to project the share of the urban population living in cities above one million, m_t. We start from the equation:

$$m_t = \frac{x_{t,\max}^{1-1/\alpha_t} - z^{1-1/\alpha_t}}{x_{t,\max}^{1-1/\alpha_t} - x_{t,\min}^{1-1/\alpha_t}} \,. \qquad [2]$$

Herein, α_t is the empirical rank-size slope, $x_{t,\min}$ and $x_{t,\max}$ are the lower and upper bounds on the sizes of a country's cities, and $z = 1$ million. As noted in the main text, the key challenge in evaluating equation Eq. (2) is to set the upper bound $x_{t,\max}$.

Our approach is to assume that $x_{t,\max}$ is proportional to the total urban population of a country U_t, i.e., $x_{t,\max} = \omega \cdot U_t$ where ω is calibrated using historical data. For each country, we grid-search $\omega \in (0.1, 2)$ and choose the value that minimizes the maximum absolute deviation between our model's estimates m_t and the observed share $\hat{m}_t$ during the period 1975-2025,

i.e., $\omega = \arg\min_{\omega\in(0.1,2)}(\max_{t\in[1975,2025]}|\hat{m}_t - m_t(\omega)|)$. Figure S6A shows that this calibration yields a strong fit between the estimated and observed share, with a mean absolute error $|m_t - \hat{m}_t|$ of 0.046.

For regional analysis, we calculate the aggregate share m_{rt} for a region r by taking the weighted average of country-level shares, where weights are based on each country's total urban population U_{ct}:

$$m_{rt} = \sum_{c\in r} \frac{U_{ct}}{\sum_{c\in r} U_{ct}} m_{ct} \ . \qquad [3]$$

At the regional level, the correlation between our estimated shares and the observed data is even stronger, with a mean absolute error below 0.01 (Fig. S6B).

C. Deriving interaction between rank-size slope and scaling exponent. Here, we derive an equation that describes how city-level scaling laws interact in the aggregate with the distribution of population (see also (1)). We begin with two assumptions:

1. A country has N cities, and their sizes (or populations) S_i follow a power-law distribution with exponent α, meaning that the share of cities larger than size x is proportional to $x^{-1/\alpha}$
2. An outcome Y scales super-linearly with city population: $Y_i \propto S_i^\theta$, where $\theta > 1$ is the urban scaling exponent.

From these assumptions, our goal is to relate the total urban population U to the aggregate value of the outcome Y_{agg}:

$$U = \sum_{i=1}^{N} S_i \qquad Y_{\text{agg}} = \sum_{i=1}^{N} Y_i = \sum_{i}^{N} S_i^\theta \qquad [4]$$

We do so by analyzing the scaling behavior of these two sums with the number of cities N, as described by two important statistical theorems:

1. Law of Large Numbers (LLN): A sum of power-law distributed random variables with exponent α *smaller* than one scales linearly with the number of terms.
2. Generalized Central Limit Theorem (GCLT): A sum of power-law distributed random variables with exponent α *greater* than one is dominated by the largest term and scales as N^α.

We start by noting that if the city size distribution follows a power-law with exponent α, then the outcome Y follows a power-law distribution with exponent $\alpha\theta$. From this observation, and the two theorems above, we deduce three distinct regimes are possible depending on whether these exponents are greater or less than 1.

1. When $\alpha < 1$ and $\alpha\theta < 1$, the LLN applies to both sums:
$$Y_{\text{agg}} \propto N \ , \quad U \propto N \quad \Rightarrow \quad Y_{\text{agg}} \propto U \ . \qquad [5]$$
2. When $\alpha < 1$ and $\alpha\theta > 1$, the LLN applies to U, while the GCLT applies to Y_{agg}:
$$Y_{\text{agg}} \propto N^{\alpha\theta} \ , \quad U \propto N \quad \Rightarrow \quad Y_{\text{agg}} \propto U^{\alpha\theta} \ . \qquad [6]$$
3. When $\alpha > 1$ and $\alpha\theta > 1$, the GCLT applies to both sums:
$$Y_{\text{agg}} \propto N^{\alpha\theta} \ , \quad U \propto N^{\alpha} \quad \Rightarrow \quad Y_{\text{agg}} \propto U^{\theta} \ . \qquad [7]$$

These asymptotic results show that more top-heaviness (a larger α) amplifies aggregate outcomes, with the effect being particularly strong in the intermediate regime $\alpha < 1$ and $\alpha\theta > 1$. The apparent vanishing of this amplification at extreme values of α is an artifact of this asymptotic approach. In any finite system, concentrating population always amplifies outcomes due to the convexity of the scaling function. Thus, for a constant θ (but see (2)), the amplifying effect of concentration is better understood as following an inverse-U shape: it is strongest in the intermediate regime and diminishes, but remains positive, at the extremes.

3. Hyperparameter robustness

In this section, we discuss the robustness of our results to changes in the hyperparameters used to conduct our analysis. We discuss robustness separately for the global and US data.

A. Global data. In the global data, our key hyperparameters are the degree-of-urbanization threshold, the city population threshold, and the country filtering conditions. To analyze robustness, we replicate Table 2 from the main text, which summarizes succinctly the core results underpinning most of the paper's conclusions: the weakening growth advantage of large cities.

A.1. Degree-of-urbanization threshold. The degree-of-urbanization threshold sets the minimum level of urbanity for a grid cell to be considered "urban". In the smod dataset of the GHSL, each cell is assigned a degree-of-urbanization imputed from built-up area estimates derived from satellite imagery and population estimates derived from censuses. The GHSL defines the following degrees-of-urbanization:

30 Urban centre, 23 Dense urban cluster, 22 Semi-dense urban cluster, 21 Suburban

13 Rural cluster, 12 Low density rural, 11 Very low density

Independent \ Dependent	Size-growth slope	
Urban population share	-0.055***	-0.043***
	(0.004)	(0.012)
Country fixed effect	No	Yes
Observations	810	810
R^2	0.199	0.572

Degree-of-urbanization threshold 22

Independent \ Dependent	Size-growth slope	
Urban population share	-0.084***	-0.091***
	(0.009)	(0.033)
Country fixed effect	No	Yes
Observations	207	207
R^2	0.281	0.441

Degree-of-urbanization threshold 23 *p<0.1; **p<0.05; ***p<0.01

Table S1. Robustness of results to variations in the degree-of-urbanization hyperparameter.

In the main paper, we set the degree-of-urbanization threshold to semi-dense urban cluster or more (≥ 22). This choice is dictated by the desire to balance two opposing requirements. On the one hand, we want to include as many cities as possible, and have a size spectrum that is as broad as possible. On the other hand, we want to ensure that distinct cities remain separate entities. A smaller threshold, such as 21 ("Suburban"), causes cities in densely populated regions (e.g., the Ganges and Yangtze river deltas) to merge into a single, massive agglomeration. Stricter thresholds, such as 23 ("Dense urban cluster") or 30 ("Urban Centre"), lead to the exclusion of small cities. Our exploration of the data suggests a threshold at 22 strikes the best balance. However, our results are robust to other threshold choices. Table S1 shows that the direction and statistical significance of our findings remain unchanged with thresholds of 23 and 30.

A.2. City population threshold. The city population threshold dictates the minimum population required for a contiguous cluster of urban cells to be defined as a "city". In the main paper, we chose 5,000 as the value of this threshold. This choice is motivated by two factors. First, it aligns with the GHSL methodology, which begins its urban classification at this population level. Second, empirical analysis of the city size distribution shows a structural break around 5,000 inhabitants, suggesting data for smaller settlements may be incomplete.

The results are robust to changes in this threshold. Table S2 shows that using population thresholds at 10,000 and 100,000 yields negatively signed coefficients similar to those in the main text. All coefficients are significant, except for that of the regression with country fixed effects at a threshold of 10,000. This loss of significance is due to a single outlier (Angola). Excluding Angola re-establishes the significance of the coefficient.

A.3. Country filtering conditions. We apply two filtering rules to determine countries to include in our analysis. The first is a minimum threshold for the number of cities in a country (which we set to 50), and the second is the manual exclusion of countries with data quality issues (Nepal and Myanmar).

Independent \ Dependent	Size-growth slope	
Urban population share	-0.040***	-0.023
	(0.005)	(0.015)
Country fixed effect	No	Yes
Observations	576	576
R^2	0.099	0.471

City population threshold 10,000

Independent \ Dependent	Size-growth slope	
Urban population share	-0.044***	-0.061***
	(0.005)	(0.014)
Country fixed effect	No	Yes
Observations	567	567
R^2	0.130	0.526

City population threshold 10,000 (No Angola)

Independent \ Dependent	Size-growth slope	
Urban population share	-0.091***	-0.074**
	(0.012)	(0.036)
Country fixed effect	No	Yes
Observations	81	81
R^2	0.439	0.617

City population threshold 100,000 $^{*}p<0.1$; $^{**}p<0.05$; $^{***}p<0.01$

Table S2. Robustness of results to variations in the city population threshold.

The minimum threshold for the number of cities in a country is chosen to ensure that our analysis of national city size distributions is meaningful. The growth advantage of large cities and the top-heaviness of the city size distribution have little relevance in countries with only a handful of cities (e.g., Singapore). The choice of 50 is a reasonable, albeit arbitrary, lower bound. Nepal and Myanmar were excluded due to data quality issues observed in our visual exploration of the data.

Our findings are robust to both filtering criteria. Specifically, Table S3 shows that our analysis replicates with a minimum threshold for the number of cities at 30 and 100, as well as including Nepal and Myanmar in the sample.

B. US data. The key hyperparameter of the US analysis is the urban threshold: the minimum population required for a cell to be classified as urban. Setting this parameter requires balancing two competing issues across a long historical period. On the one hand, low thresholds cause large cities to merge, particularly in later years when census place populations are large. On the other hand, high thresholds exclude small cities, especially in the early years, when census place populations are small. Our threshold at 50 people is a compromise: it allows us to have small cities (of 5,000 people) across the whole time frame, while limiting mergers of large cities.

As we vary the threshold, the major difference is mechanical and concentrated in the early period. When we raise the threshold to 200, many small cities drop from the sample (Fig. S7C), leaving only a few large cities. This makes the estimated size–growth slope for that period noisy (green line, Fig. S7D). This attrition is smaller in later periods, when city populations are larger (Fig. S7C).

Apart from this truncation and its downstream noise, the results are robust to the choice of threshold. The size–growth curves have essentially the same shape across cutoffs (Fig. S7A-B). Further, the main pattern identified in the main paper — the weakening growth advantage of large cities — reproduces at different thresholds, with the caveat of extra noise in the early period at the 200 threshold (Fig. S7D). The findings also replicate with a time-varying threshold, which preserves the sample of small cities in the early period while reducing the mergers of large cities in the late period (Fig. S7D Inset).

Independent \ Dependent	Size-growth slope	
Urban population share	-0.050***	-0.052***
	(0.003)	(0.011)
Country fixed effect	No	Yes
Observations	999	999
R^2	0.171	0.549

Countries with at least 30 cities

Independent \ Dependent	Size-growth slope	
Urban population share	-0.046***	-0.055***
	(0.004)	(0.011)
Country fixed effect	No	Yes
Observations	594	594
R^2	0.188	0.605

Countries with at least 100 cities

Independent \ Dependent	Size-growth slope	
Urban population share	-0.041***	-0.037***
	(0.004)	(0.013)
Country fixed effect	No	Yes
Observations	909	909
R^2	0.102	0.528

Base sample + Myanmar and Nepal $^{*}p<0.1$; $^{**}p<0.05$; $^{***}p<0.01$

Table S3. Robustness of results to variations in the sample of countries.

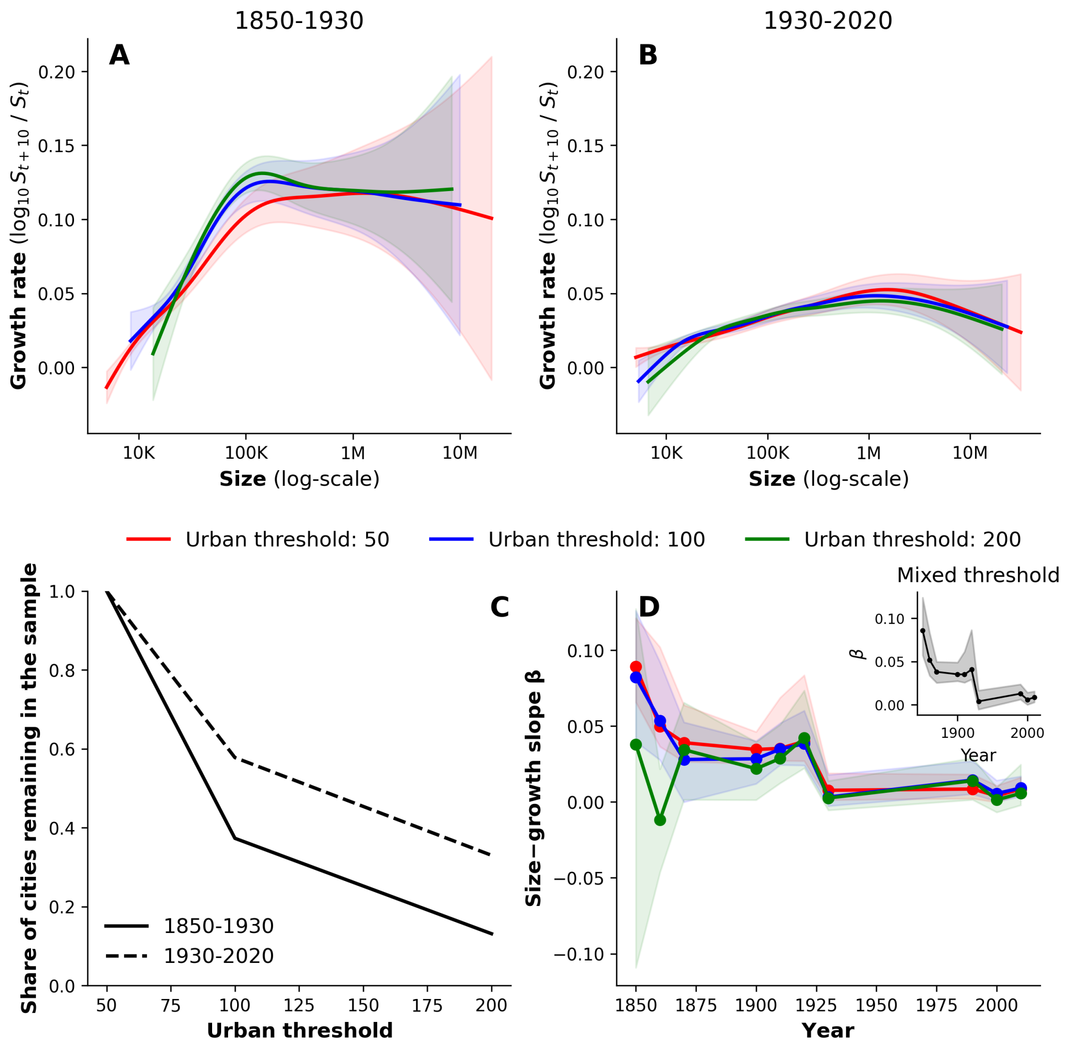

Fig. S7. Robustness to variation in the urban threshold for US data. **(A-B)** The shape of the size-growth curve for different urban threshold choices. **(C)** Share of cities remaining in the sample by threshold: raising the threshold from 50 to 200 removes over 80% of cities in the early period (1850-2020). This helps explain the instability of the green curve in (D). **(D)** Historical trends in β for different urban thresholds. (Inset) Historical trends in β using a mixed threshold (50 for 1850-1930, at 200 for 1930-2020) .

4. Other robustness tests

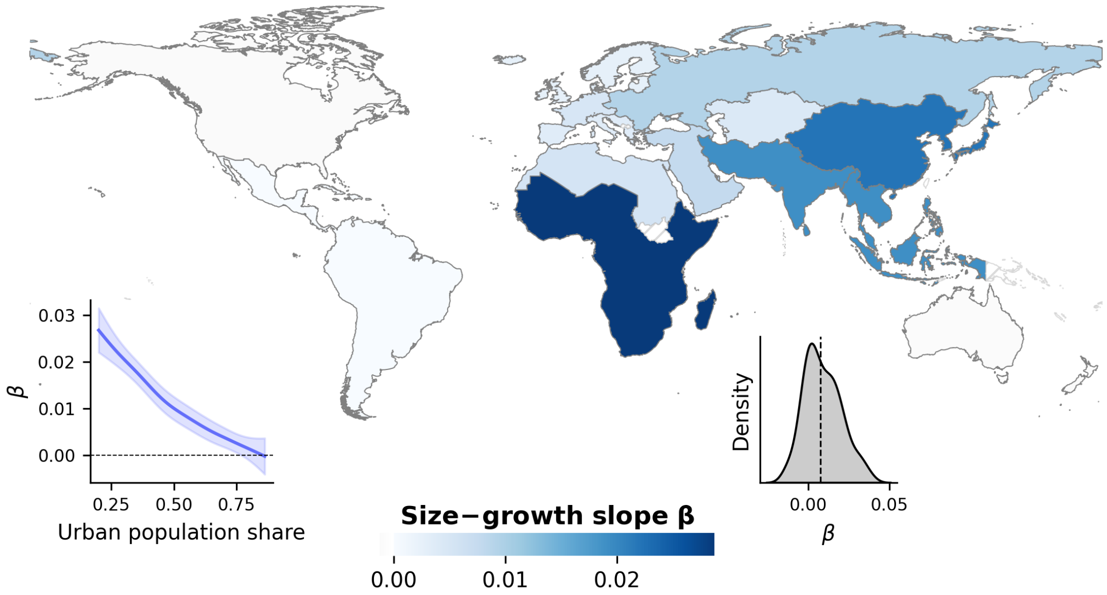

Fig. S8. Robustness of results to aggregation at the level of UN M49 sub-regions. The map shows the mean size-growth slope β by sub-region, averaged over the 1975-2025 period (cf. Figure 1C in the main text). For each sub-region-year, β is estimated by pooling cities within the sub-region and fitting the same penalized cubic B-spline used in the main text ($\lambda = 100$). Hatched areas indicate no data. The left inset shows the relationship between β and the sub-regional urban population share, using a penalized cubic B-spline fit (cf. inset of Figure 2A in the main text). The right inset shows a kernel density estimate of the distribution of β across sub-region-years (dashed line = median).

A. Choice of national borders. In the main paper, we define urban systems using national borders for three reasons. First, national borders constrain migration flows and infrastructure investment, which are central mechanisms of differential urban growth (3). Second, national borders are the most commonly used delineations of urban systems in the literature on city-size distributions, making our work easier to compare with earlier studies. Third, data and projections about population and urban population share are typically reported at the national level.

We recognize that this approximation is not exact, because some urban systems extend across borders. For this reason, we provide further evidence that the core pattern we identify—a declining growth advantage of large cities as urbanization progresses—holds qualitatively at different scales. In the main text, we show this across four broad macro-regions (Asia, Europe, Africa, and the Americas). Here, we show it across the UN M49 sub-regions.

The UN M49 classification defines 16 geographical sub-regions that are more homogeneous than continents but broader than individual countries (see the map in Fig. S8). After filtering out sub-regions with fewer than 50 cities (Polynesia and Melanesia), we are left with 14 sub-regions, yielding 126 sub-region-year observations. For each sub-region-year, we pool cities from the countries in the sub-region and estimate the size-growth slope β with the same penalized cubic B-spline procedure used in the main text ($\lambda = 100$). The sub-regional urban population share is computed as a population-weighted average of the urban population shares of the countries within the sub-region.

Fig. S8 shows that the sub-regional pattern closely resembles the country-level one. The average β is high in Sub-Saharan Africa, Southern Asia, and Eastern Asia, while it is low in Western Europe, South America, and North America. The negative association with the urban population share remains clear: a pooled OLS regression of β_{rt} on the sub-regional urban population share* u_{rt} yields a negative slope of -0.0363, with a 95% confidence interval of $[-0.044, -0.029]$ and $R^2 = 0.436$. The coefficient is somewhat smaller in magnitude than in the pooled country-level regression (-0.0363 vs. -0.049), but the fit is substantially better ($R^2 = 0.436$ vs. 0.168). This suggests that the relationship becomes less noisy at this intermediate spatial scale.

B. Urban population share data. In the main text, we use the urban population share data from Chen et al. (4). A drawback of this dataset is that it relies on national statistical agencies to define "urban", introducing potential cross-country inconsistencies. Here, we complement the main text results with a robustness check. We replicate Table 2 from the main text, using an alternative urban population share measure from the History Database of the Global Environment (HYDE) (5). HYDE is more consistent with our city definition because its urban population share estimates are derived from spatially explicit grids that combine historical demographic estimates with land-use allocation algorithms. Table S4 shows that our main findings are robust to this alternative measure of urban population share.

*We compute this share by taking the population-weighted average of the urban population shares of countries within the sub-region.

Independent \ Dependent	Size-growth slope	
Urban population share	-0.051***	-0.048***
	(0.004)	(0.011)
Country fixed effect	No	Yes
Observations	891	891
R^2	0.172	0.545

*p<0.1; **p<0.05; ***p<0.01

Table S4. Association between size-growth slope and urban population shares using a different dataset ((5)) to define the urban population shares of countries.

5. Suburbanization

A natural candidate mechanism to explain the declining growth advantage of large cities is suburbanization. As urbanization progresses, population growth may shift away from the urban core and toward suburban satellites, reducing the core's growth rate while increasing that of the suburbs. Since suburbs tend to be small and cores large, this reallocation of growth would flatten the size–growth curve and could therefore be an important contributor to the decline in β.

We examine this mechanism in two steps. First, we focus on the United States, comparing our geographic city definitions with functional urban areas to directly isolate the role of suburbanization. Second, we construct a simple gravity-based approximation of functional urban areas from satellite-based morphological clusters (hereafter, called "base clusters") and apply it globally to extend our USA-related results.

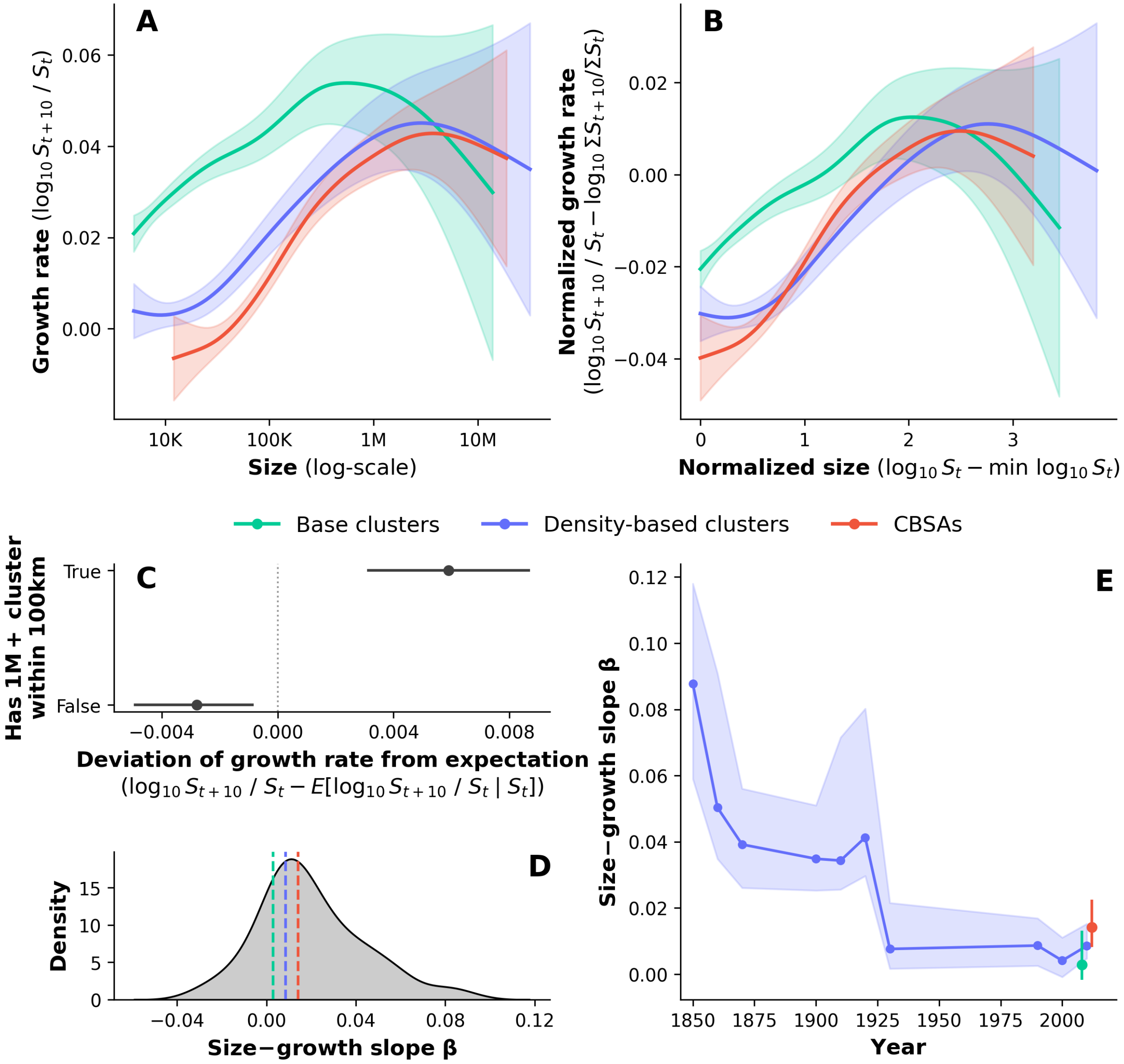

Fig. S9. Suburbanization as a candidate mechanism for the decline of the size-growth slope in the USA. **(A)** Size-growth curves for the USA in 2010-2020 under three city definitions: base clusters from the Global Cities Dataset, density-based clusters from the USA Cities dataset, and Core-Based Statistical Areas (CBSAs). For each definition, population growth is measured within stable decade-specific boundaries obtained with the procedure in Methods 4.2. The curves are estimated using penalized cubic B-splines (Methods 4.3.2). **(B)** The same curves after horizontal and vertical translation to compare their shapes. The x-axis is translated by subtracting the minimum observed log-size of each definition. The y-axis is translated by subtracting the average log-growth of the urban population, $\log_{10}(\sum_i S_{i,t+10}/\sum_i S_{i,t})$. **(C)** Average deviation from the expected growth among USA clusters below 1M inhabitants in 2010, split by whether they are located within 100km from a 1M+ cluster or not. Expected growth is the value of the size-growth curve at the cluster's size. **(D)** Distribution of country-level size-growth slopes β in the Global Cities Dataset for 2010-2020; vertical lines indicate the USA estimates from the three definitions. **(E)** Historical evolution of the USA size-growth slope β in the density-based data, with the 2010-2020 estimates from the base-cluster and CBSA definitions overlaid.

A. Definition comparison for the USA. We compare three city definitions over the period 2010-2020: (i) base clusters from the Global Cities Dataset; (ii) density-based clusters from the USA Cities dataset; and (iii) Core-Based Statistical Areas (CBSAs), where the official functional definition is based on commuting ties. For all three, we estimate growth using the same boundary-matching procedure within the City Clustering Algorithm (CCA) framework described in Methods 4.2: we match 2010 clusters to their 2020 counterparts, construct a stable city boundary by taking the union of the connected components of the matching graph, and measure population change within this boundary. CBSAs were introduced in 2003, making 2010-2020 the first full census decade for which this comparison is possible. We avoid back-projecting modern functional boundaries into earlier periods because it introduces selection bias. In fact, defining a historical sample based on 2020 functional areas systematically selects for cities that grew rapidly. Small settlements that stagnated fail to meet the threshold for modern CBSA designation and are excluded, whereas those that grew rapidly meet the threshold and thus are included. Consequently, back-projecting boundaries inflates the growth rates of smaller cities.

Figure S9A shows that the density-based and CBSA curves are already fairly similar, while the base-cluster curve is visibly higher for small cities. To compare the *shape* of the curves rather than their levels, we translate each curve horizontally and vertically (Fig. S9B). Specifically, for each city i, we plot

$$\tilde{s}_{it} = \log_{10} S_{it} - \min_j \log_{10} S_{jt}, \qquad \tilde{g}_{it} = \log_{10}\left(\frac{S_{i,t+10}}{S_{it}}\right) - \log_{10}\left(\frac{\sum_j S_{j,t+10}}{\sum_j S_{jt}}\right) . \qquad [8]$$

Here, j indexes cities within a given definition. This operation aligns the lower end of the size spectrum and removes the definition-specific average growth level, but it does not rescale the curve or change its slope. After this alignment, the density-based and CBSA curves largely overlap, whereas the base-cluster curve remains higher on the left-hand side.

This pattern is consistent with suburbanization. If small clusters near a large city grow faster than small clusters far from a large city, integrating these clusters with their larger neighbors would shift the curves in exactly this way. To test this directly, we focus on USA base clusters with fewer than 1M inhabitants. We split these clusters into two groups: those located within 100 km of a 1M+ cluster and those that are not. We then measure whether these small clusters grow faster or slower than expected, given their size, where the expected growth is the fitted value of the USA 2010–2020 size–growth curve at the cluster's log-size. For cluster i, the deviation from expectation is

$$\delta_i = \log_{10}\left(\frac{S_{i,t+10}}{S_{it}}\right) - \hat{f}\left(\log_{10} S_{it}\right) . \qquad [9]$$

Here, $\hat{f}$ is the fitted national size-growth curve. Positive values indicate that the cluster grew faster than typical for clusters of that size. Negative values indicate the opposite. Figure S9C shows a statistically significant positive result for the suburbanization channel: small clusters near a 1M+ city have positive average deviations, while small clusters far from such cities have negative average deviations.

The remaining question is how much of the size-growth slope trends identified in the main text can be explained by this mechanism. Figure S9D-E suggests it has limited explanatory power, at least in the US. In the historical USA series, the density-based estimate of β falls from roughly 0.09 in 1850 to roughly 0.01 by 2010, whereas the gap across the three 2010-2020 definitions is of the order of 0.01 or less (Fig. S9E). Similarly, when the 2010-2020 USA estimates are placed against the cross-country distribution of β in the Global Cities Dataset during that same decade, the spread across definitions occupies only a small part of that distribution (Fig. S9D). Thus, empirical evidence for the USA indicates that suburbanization is present and measurable, and that it pushes β in the expected direction, but that its magnitude is small relative to other forces.

B. Gravity-based approximation of functional urban areas. To extend the same analysis beyond the USA, we construct a simple gravity-based approximation of functional urban areas from the base clusters. The idea is to use a gravity-based heuristic to decide whether to absorb a suburban satellite into a large metropolitan area nearby. For each ordered pair of base clusters (c_1, c_2), let $d(c_1, c_2)$ denote the distance between their geometries, S_c the population of cluster c, and $A(c)$ its area. We define the *pull* of c_1 on c_2 and the *self-pull* of a cluster c:

$$\text{pull}(c_1 \to c_2) = \frac{S_{c_1}}{d(c_1, c_2)}, \qquad \text{self-pull}(c) = \frac{S_c}{\sqrt{A(c)}} . \qquad [10]$$

In the self-pull equation, the term $\sqrt{A(c)}$ acts as a proxy for the characteristic internal distance of the cluster. Because a settlement's radius is proportional to the square root of its area, this term captures the expected distance between random inhabitants within the city, thereby allowing for a comparison with external distances. For each base cluster c, we identify the neighbor exerting the largest pull. If this largest external pull exceeds self-pull(c), the base cluster is reassigned to that neighbor; otherwise, it remains separate. This procedure yields "augmented clusters". Intuitively, these augmented clusters are the base clusters merged with smaller nearby base clusters, on which they exert a strong pull. Empirically, the mean distance between a merged satellite and its central cluster is 25 km (interquartile range: 5 to 40 km).

To verify the effectiveness of this gravity-based method in approximating functional urban areas, we compare the partition of the base clusters induced by the augmented clusters with that induced by CBSA membership. Specifically, we assign each base cluster c two labels:

1. $u(c)$ is the CBSA with which c has the largest area of overlap.

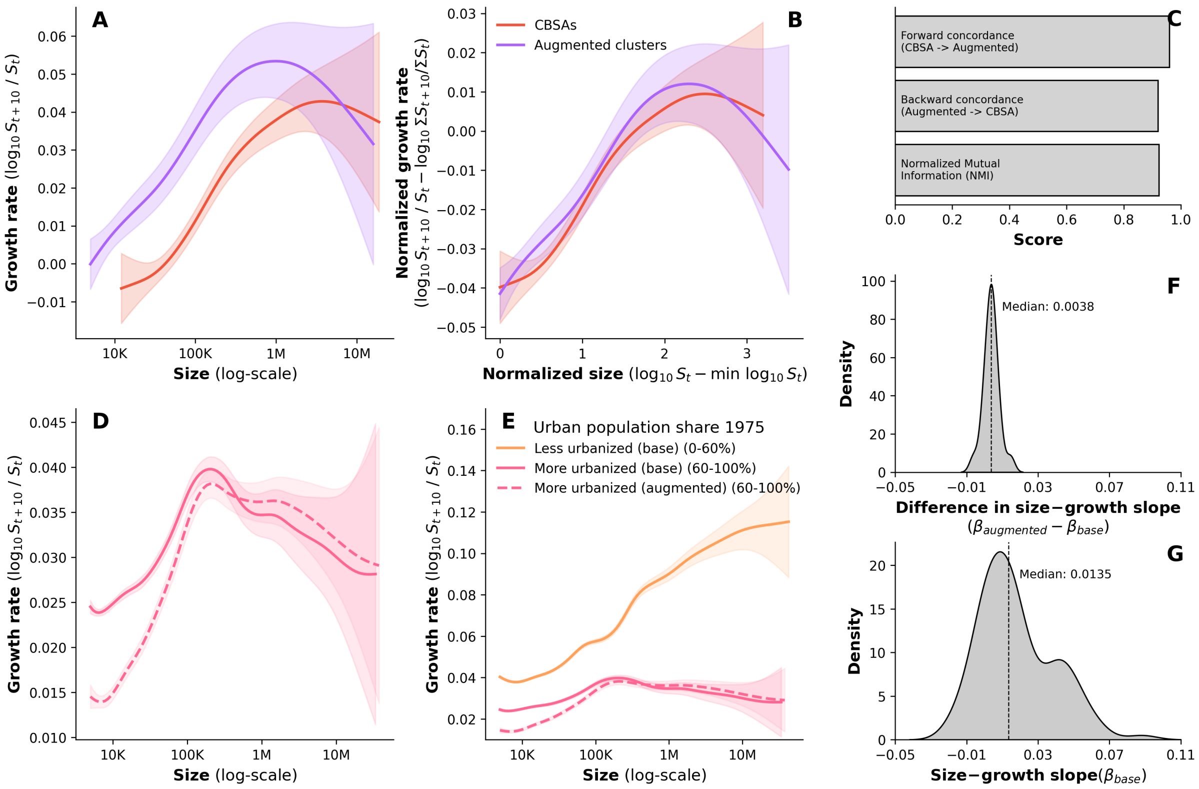

Fig. S10. Gravity-based approximation of functional urban areas and the magnitude of the suburbanization channel. **(A)** Size-growth curves for CBSAs and gravity-based augmented clusters in the USA 2010-2020. **(B)** Size-growth curves in (A) translated as in Fig. S9B. **(C)** Validation of gravity-based grouping against CBSAs using forward concordance, backward concordance, and normalized mutual information. **(D)** Size-growth curves for the more-urbanized group under the base-cluster and augmented definitions. Curves are estimated as in Methods 4.3.2. **(E)** Size-growth curve for the less-urbanized group under the base-cluster definition, and for the more-urbanized group under both the base-cluster and augmented definitions. **(F)** $\beta_{\text{augmented}}$ and β_{base} denote the size-growth slopes estimated using the augmented and base-cluster definitions. The figure shows the distribution of $\beta_{\text{augmented}} - \beta_{\text{base}}$ across countries in the more-urbanized group. For comparison, we set the same x-range as in (G). **(G)** Distribution across all countries of β_{base}. With a mean difference of 0.0037 in (F) and a standard deviation of 0.0206 in (G), the standardized difference represents 0.18 standard deviations of the global base distribution.

2. $v(c)$ is the augmented cluster with which c has the largest area of overlap.

We then compute forward concordance, backward concordance, and normalized mutual information on these labels, weighting by population. Forward concordance measures the probability that two clusters that share the same CBSA label ($u(c)$) also share the same augmented cluster label ($v(c)$). Backward concordance measures the reverse: the probability that two clusters with the same augmented cluster label share the same CBSA label. Normalized mutual information (NMI) calculates the overall statistical dependence between the two partition systems. All three metrics range from 0 (completely independent partitions) to 1 (perfectly identical partitions). The resulting scores are high: forward concordance is 0.92, backward concordance is 0.96, and NMI is 0.92 (Fig. S10C), implying a close correspondence between the partitions. This correspondence is also visible in a simpler validation: the augmented clusters generate a size-growth curve that is similar in shape to the CBSA curve (Fig. S10A-B; this is particularly visible after translation).

Given this validation, we apply the same procedure to the countries in the "more-urbanized group" defined in the main text (60-100% urban population share in 1975), where suburbanization is likely to be most pronounced. In Fig. S10D, we compare the size-growth curve estimated using augmented clusters to that estimated using the base clusters. As in the USA (Fig. S10B), we observe that the difference between the two curves concentrates on the left-hand side: small augmented clusters grow slower than small base clusters, consistent with suburbanization. However, this difference is small compared to the difference in size-growth curves between our more-urbanized and less-urbanized country groups (Fig. S10E). Quantitatively, the mean difference in size-growth slopes between the two definitions ($\beta_{\text{augmented}} - \beta_{\text{base}}$) represents a shift of only 0.18 standard deviations of the global distribution of base slope.

We re-estimate size-growth slopes using augmented clusters ($\beta_{\text{augmented}}$) and compare them with the original size-growth slopes estimated using base clusters (β_{base}). The country-level distribution of $\beta_{\text{augmented}} - \beta_{\text{base}}$ is shifted slightly to the right, with a median of about 0.004 (Fig. S10F). This is consistent with suburbanization making the base-cluster slopes flatter. At the same time, the flattening is small: the differences $\beta_{\text{augmented}} - \beta_{\text{base}}$ span just a fraction of the variation in β_{base} across countries (Fig. S10G). We also re-ran the regression in Table 2, replacing β_{base} with $\beta_{\text{augmented}}$ for countries in the more-urbanized group, and obtained similar results (Table S5).

Taken together, these results show that suburbanization is a real and detectable part of the urban life cycle. However, it is

quantitatively too small to explain the overall decline in the growth advantage of large cities. The life cycle documented in the main text must therefore reflect deeper forces that go beyond the spatial redistribution of population within metropolitan areas.

Independent \ Dependent	Size-growth slope	
Urban population share	-0.048***	-0.056***
	(0.004)	(0.012)
Country fixed effect	No	Yes
Observations	810	810
R^2	0.141	0.531

*p<0.1; **p<0.05; ***p<0.01

Table S5. Association between country urban population share and size-growth slope β. We use augmented clusters to estimate β for countries in the "more-urbanized group" (60-100% urban population share in 1975) and the base clusters to estimate β for the "less-urbanized group" (0-60% urban population share in 1975).

References

1. A Gomez-Lievano, H Youn, LMA Bettencourt, The Statistics of Urban Scaling and Their Connection to Zipf's Law. *PLOS ONE* **7**, e40393 (2012).
2. HV Ribeiro, M Oehlers, AI Moreno-Monroy, JP Kropp, D Rybski, Association between population distribution and urban GDP scaling. *PLOS ONE* **16**, e0245771 (2021).
3. V Verbavatz, M Barthelemy, The growth equation of cities. *Nature* **587**, 397–401 (2020).
4. S Chen, et al., Updating global urbanization projections under the Shared Socioeconomic Pathways. *Sci. Data* **9**, 137 (2022).
5. K Klein Goldewijk, History database of the global environment (HYDE) 3.3 (2023).
